# The evolutionary game analysis of the decision-making behavior of live streaming stakeholders under the value co-creation

**DOI:** 10.1371/journal.pone.0291453

**Published:** 2023-09-22

**Authors:** Shanshan Jia

**Affiliations:** Shijiazhuang University of Applied Technology, Shijiazhuang, China; Sichuan Agricultural University, CHINA

## Abstract

Integrity-linked issues have become increasingly serious and attracted considerable attention regarding the sustainability of live streaming; therefore, live streaming anchors and suppliers have become increasingly concerned about their ongoing marketing. Moreover, streaming platforms are also beginning to focus on sustainable development. Determining how live streaming anchor behavior, supplier behavior, and streaming platform strategic decision affect live streaming is essential for achieving the healthy and sustainable development of live streaming ecosystems. However, the game among live streaming anchor behavior, supplier behavior, and streaming platform strategic decisions, which the live streaming ecosystem strongly depends on, has attracted little attention. This study constructed a game model of value co-creation evolution in live streaming ecosystems, with the live streaming anchors, suppliers, and streaming platforms as the main subject. The evolutionary stable strategies of the game were analyzed, and the influence of typical factors on the system was discussed. The results showed that the best evolutionary stability strategy is that live streaming anchors and suppliers choose honesty, and streaming platforms have strict regulation, which is the optimal strategic objective for value co-creation. The appropriate rewards and punishments inherent in a streaming platform can help regulate the behavior of the live streaming anchor and supplier; specifically, great rewards and severe punishments of the streaming platform can be applied to incentivize the honest behavior of the live streaming anchor and supplier. This study provides insights for designing appropriate policies for live streaming platforms to promote sustainable development.

## 1. Introduction

With the rapid development of technologies such as artificial intelligence, virtual reality, and big data, live streaming e-commerce continues to transcend barriers faced by other sales channels (i.e., spatiotemporal and information limitations) [1], and its overall structure supports the efficient trade of a wide array of goods and services. In August 2022, the China Internet Network Information Center (CNNIC) released the 50^th^ Statistical Report on the Development Status of China’s Internet; as of June 2022, the number of domestic live streaming platform users exceeded 716 million, accounting for approximately 68.1% of the overall Internet users. The number of live e-commerce users was 469 million, an increase of 5.33 million compared to the previous year [2]. In recent years, the global economy has been drastically affected by the COVID-19 epidemic. This epidemic-driven economic downturn (combined with home isolation policies) fast-tracked the development of the Internet economy, especially within the context of platforms [3]. Live streaming enables consumers to purchase their favorite products at home, promote local ethnic customs, increase the sales channels of unique agricultural products, promote industrial revitalization, increase job opportunities, and promote economic development.

However, the recent and rapid development of live streaming platforms has highlighted weaknesses that could threaten the continued growth of these live streaming platforms. For example, according to the Beijing Consumer Association’s live streaming consumer survey report, among 30 samples of live streaming experience, 9 were suspected of having problems with the disclosure of license information, 3 samples were suspected of having problems with false promotion, and 1 sample was not able to enforce the “an unconditional 7 days return policy.” Based on these issues, it is important to research the problems of live streaming industry and propose appropriate governance measures that protect citizens’ legal rights and guide the sustainable development of live streaming industry.

Limited research has been conducted on the management issues of live streaming platforms. Most researchers have combined psychology [4], communication [5], and other related fields to analyze these live streaming platforms qualitatively. However, most qualitative research methods are greatly impacted by subjectivity and biases. Researchers have also used evolutionary game theory to study this issue [1]. However, this quantitative approach is based on the assumption of a "rational broker" [6].

There are three main subjects of live streaming industry. The first is the live streaming anchor that delivers promotion for goods. The live streaming anchor mainly cooperates with suppliers and receives consumer rewards to obtain revenue. The second subject is the supplier who signs a contract with the live streaming anchor to improve their sales online, and whose primary revenue is generated via the sale of the goods. The third subject is a live streaming platform that provides a promotional space for live streaming anchors with goods and obtains benefits through profit draw and consumer reward share. Typical examples are Jitterbug and Racer. To attain sales outcomes, collusion may occur between the live streaming anchor and supplier to maximize benefits using false promotion. This collusion can negatively affect social credit while jeopardizing the legitimate interest of consumers. Therefore, analyzing the game between the live streaming anchor, supplier, and live streaming platform is important for purifying the live streaming network environment, maintaining social credit, and protecting consumer rights.

Although live streaming ecosystems strongly depend on live streaming anchor behaviors, supplier behaviors, and strategic decisions of the live streaming platform, the game between these three parties has not been sufficiently investigated. Therefore, the purpose of this study was to utilize evolutionary game theory to explore the impact of live streaming anchors’ behavior, suppliers’ behaviors, and live streaming platform decision-making on the live streaming ecosystem to determine the factors that influence the ecosystem. The results of this study form an important basis for the game parties to maximize utility and for the live streaming platform to develop effective reward and punishment policies.

## 2. Literature review

### 2.1. Study on live streaming platforms

The traditional e-commerce model, in which consumers can only browse one way and read static product content online to obtain product information, leads to uncertainties and risk perceptions about the purchase of products, thereby affecting the purchase rate [7, 8]. In contrast, live streaming platforms create a virtual live space for live streaming anchors to establish virtual social relationships, achieve instant interaction, and offer consumers variety of content in a relaxing and entertaining atmosphere [9]. Previous studies on live streaming platforms mostly focused on two aspects. First, they considered regulatory strategies and governance countermeasures as an entry point to elaborating on the necessity of regulatory strategies for live streaming platforms [10, 11]. From a stakeholder perspective, Chen et al. [1] discussed the intrinsic mechanism of live Internet broadcasting governance under the participation of multiple parties and proposed corresponding network governance strategies in conjunction with game theory analysis. From a content supervision perspective, Li et al. [12] proposed a system to intelligently monitor the misconduct of illegal live broadcasts, which entails the manual review of live streaming content. From the perspective of live streaming platforms, a method to improve user experience was proposed that entails resolving specific problems, such as the broadcast delay between video recording and viewing on Internet live streaming platforms and the synchronization of playback between clients. Two identified reasons for the lack of profitability of live streaming platforms are a mismatch between the ratio of contracted live streaming anchors, base size of live streaming platforms, and inappropriate contracting strategy of live streaming platforms [13, 14]. Although these studies can contribute constructive opinions to support the sustainability of live streaming industry, they ignored the legitimate interest of such subjects as live streaming anchors, suppliers, and live streaming platforms. Second, they focused on the influence of elements within the live streaming room on users’ purchase intentions. In a study of factors underlying consumer purchases, well-designed live streaming platforms and rewards (e.g., virtual gifts) have been shown to stimulate consumers’ desire to purchase [15–18]. Zhou et al. [19] analyzed users’ interactions with live streaming platforms and found that the total number of words in pop-ups and social competition and the number of words related to the similarity and excitement of the pop-up positively increased the odds of users making a purchase. Class and relationship identities also affect consumer behavior [20]. When studying factors influencing user purchase behavior, convenience, user behavior, trust in merchants and live streaming anchors, and behavioral intention play an essential role in live streaming purchase behavior [3], when studying factors influencing user purchase behavior. The behavior of users has revealed a large amount of sharing among users [21]. Using the Stimulus, Organism, Response (SOR) model as a theoretical framework, some scholars have used relationship strength as theoretical evidence to conclude that user interactivity and participation behavior on live streaming platforms are curvilinearly associated [22]. Although it is helpful for brands or merchants to improve the economic benefits of e-commerce live streaming by adjusting the elements, these micro-level studies could yield better insights if they applied more systematic and dynamic approaches. Evolutionary game theory is an integrated approach that combines game theory with dynamic evolution [23, 24]; it has been widely used in new energy vehicles, photovoltaics, industrial pollution, and power equipment [25–28]. The behavioral strategy choices of live streaming anchors, suppliers, and live streaming platforms drastically influence the tripartite revenue of live streaming. Related issues have received attention and focus from various perspectives. In recent years, scholars have begun to utilize game theory to discuss the mechanism of influence inherent in e-commerce participants. Li et al. [29] introduced buyer’s feedback into the credit regulation system of e-commerce platforms, constructed a three-party evolutionary game model with e-commerce platforms, vendors, and consumers as the leading players under information asymmetry, and analyzed the influencing factors of strategy selection. Chen et al. [1] classified comments on various live streaming platforms and constructed a tripartite evolutionary game model with a live streaming platform, live streaming anchor, and consumers as principal actors. Aringhieri et al. [30] analyzed an online reputation system in e-commerce through a hybrid game and agent simulation. They constructed a model to discover the effects of buyers’ and sellers’ different behaviors related to e-commerce policies.

### 2.2. Study on the value co-creation

The value co-creation theory originated in service marketing and was first proposed by Normann and Ramirez [31]. Value co-creation is the reverse development of value co-destruction, and is a process of resource integration; if one of the parties lacks the necessary resources, value co-creation may fail, thus leading to value co-destruction [32]. There is a critical point between value co-creation and co-destruction, which can be transformed into each other. In the interaction network of multiple actors, value co-creation does not imply that value co-destruction will not occur; value co-destruction may be a stage in the co-creation process, and co-creation may eventually develop into co-destruction. The literature review indicated that value co-creation had been widely studied in several areas, including health care, social media, tourism, public services, and retail [33–37]. However, it was also found that few researchers have focused on value co-creation in the live streaming ecosystem. Therefore, to address the gap in the existing literature, this study innovatively investigated live streaming platforms from a value co-creation perspective.

### 2.3. Study on evolutionary game

Evolutionary game theory integrates game theory analysis with dynamic evolutionary processes [23, 24]. In classical game theory, participants are presumed to be completely rational and have great memory and excellent analytical skills [38]. However, in reality, participants are not completely rational, thus making the practical value of insights obtained via this method very low; therefore, this approach is limited in practice [39]. Participants with different learning and decision-making abilities are not entirely rational but are constantly learning, imitating, and optimizing their decisions. In a repeated game of competition and cooperation, players’ behavior gradually stabilizes, and strategic change can be described as strategy selection-evolution-re-evolution [40–42].

Evolutionary games are defined by competing objectives and interacting tactics, and they resemble the fundamental components of customer and business relationships [39]. Therefore, this approach has been widely used in recent years to analyze players’ strategic choices under interaction [43]. Evolutionary game theory has been extensively applied to cooperative behavior and strategy selection issues. In the live streaming ecosystem, there are interests among live streaming anchors, suppliers, and live streaming platforms, and their decisions are a dynamic process driven by profit; therefore, the evolutionary game approach is appropriate. However, many studies emphasize the importance of regulatory governance concerning live streaming platforms [1] and largely focus on games between live streaming anchors and buyers [18, 19].

To the best of our knowledge, an analysis of the factors that influence the partnership of live streaming anchors, suppliers, and live streaming platforms that considers the value co-creation in the live streaming platform ecosystem based on evolutionary game theory has not been performed. To fill this research gap, this study used tripartite evolutionary game theory to establish an evolutionary game model of the partnership among the three participants from the perspectives of value co-creation, estimated the influences of several typical factors on strategy selection by the live streaming participants, and analyzed the evolutionary processes of participants. Based on the evolutionary game theoretical analysis results, policies and approaches were suggested to realize the value co-creation of live streaming ecosystem.

## 3. Evolutionary game model

**Fig 1:**
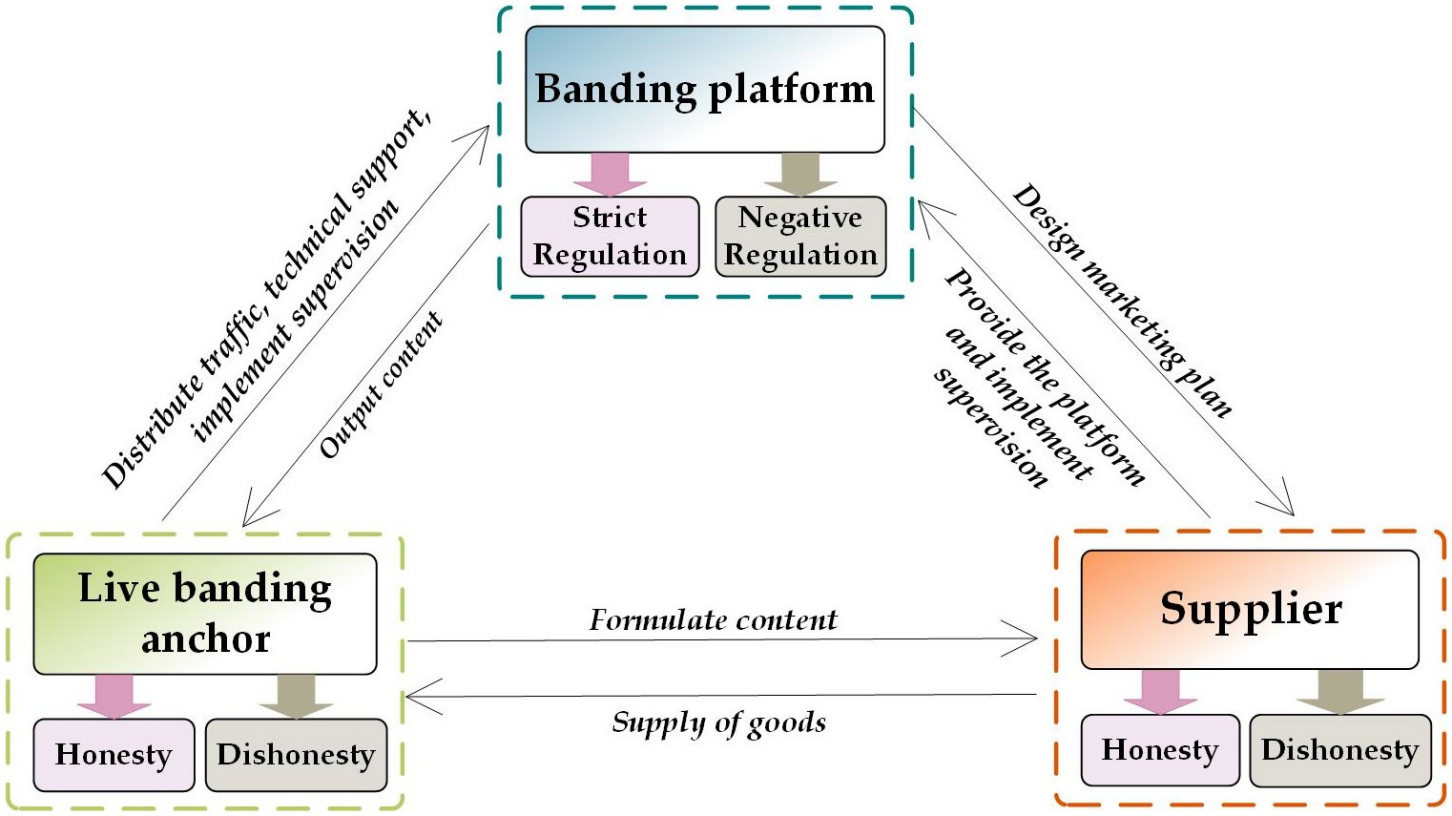
Logical relationship between the subjects of the three-party evolutionary game of live streaming.

In the context of weak supervision, uneven quality of live streaming anchors, and explosive economic growth of Internet celebrities, special rectification and planning management actions are needed to promote “live streaming with goods” to a formal state. The subjects of the live streaming include the live streaming anchor, supplier, and live streaming platform. To obtain better sales performance, collusion between the livestreaming anchor and supplier will occur to maximize benefits through false promotion. To prevent such violations, the live streaming platform must adopt appropriate policies to maintain its credibility. Therefore, this study selected the live streaming anchor, supplier, and live streaming platform to model a tripartite evolutionary game model and formulated constraints to improve the live streaming environment, maintain social credit, and maintain the sustainable development of the live streaming industry. The logical relationship of the evolutionary game among the three subjects in live streaming constructed is shown in Fig 1.

### 3.1. Model assumptions and parameter settings

Section 2.3 indicates that the evolutionary game model is applicable to the study of value co-creation in the live streaming ecosystem. Based on previous research [44, 45] and the reality of live streaming, the following assumptions were made.

**Hypothesis 1 (H1)**. “Broker” assumption. In the gaming process, the suppliers aim to maximize their interests. To expand product sales and maximize their interests, both parties may reach some agreement privately and engage in collusive behavior to promote and sell products utilizing false gimmicks and false promotion. The live streaming platform will take strict regulatory measures to limit the illegal collusion between the live streaming anchor and the supplier for its sustainable and healthy development. However, strict regulation requires regulatory costs; therefore, the regulatory behavior of the live streaming platform is probable.
**Hypothesis 2 (H2)**. “Limited rationality” assumption. All three parties are finitely rational participants, and the strategy choice evolves gradually over time to stabilize into an optimal strategy.
**Hypothesis 3 (H3)**. Strategy selection hypothesis. Each of the game’s three subjects can choose between two strategies. The strategy space of the hosts is {honesty, dishonesty}, where the probability of the hosts choosing honesty is x, the probability of choosing dishonesty is 1 − x, (0 ≤ x ≤ 1); the strategy space of the suppliers is {honesty, dishonesty}, where the probability of the suppliers choosing honesty is y, the probability of choosing dishonesty is 1 − y, (0 ≤ y ≤ 1); the strategy space of the live streaming platform is {strict regulation, negative regulation}, where the probability of the live streaming platform choosing strict regulation is z, and the probability of choosing negative regulation is 1 − z, (0 ≤ z ≤ 1).
**Hypothesis 4 (H4)**. Model parameters are assumed. To facilitate the construction of the tripartite revenue matrix and ensure the reasonableness of the parameter settings, after referring to the relevant literature [46] and the opinions of experts, and combining the actual situation of the live streaming, the parameters are set as shown in Table 1 from the perspective of cost, revenue, and loss.

**Table 1:** Parameter symbols and definitions.

Parameters	Explanatory Notes
***x***	probability of live streaming anchor choosing the “good faith” strategy, 0 ≤ *x* ≤1
***y***	probability of suppliers choosing the “good faith” strategy, 0 ≤ *y* ≤1
***z***	probability of the live streaming platform choosing the “strict supervision” strategy, 0 ≤ *z* ≤1
***Ea***	gain obtained by the live streaming anchor, *Ea* > 0
***Es***	gain obtained by the supplier, *Es* > 0
***Ep***	gain obtained by live streaming platform, *Ep* > 0
***Ca***	cost paid by the live streaming anchor when choosing honesty, *Ca* > 0
***Cs***	cost paid by the supplier when choosing honesty, *Cs* > 0
***Cp***	cost paid by the live streaming platform when it chooses to strictly regulate, *Cp* > 0
***Ia***	network externality gains under live streaming anchor honesty in the case of strict live streaming platform regulation, *Ia* > 0
***Is***	network externality gains under supplier honesty in the case of strict live streaming platform regulation, *Is* > 0
***α***	revenue distribution coefficient between live streaming platform and live streaming anchor, 0 ≤ *α* ≤ 1
***β***	revenue allocation coefficient between live streaming platform and supplier, 0 ≤ *β* ≤ 1
***λ***	reward strength, 0 ≤ *λ* ≤ 1
***λRa***	total incentive of the live streaming platform for the live streaming anchor’s honest behavior, *λRa* ≥ 0
***λRs***	total incentive of the live streaming platform for suppliers’ honest behavior, *λRs* ≥ 0
***θ***	penalty strength, 0 ≤ *θ* ≤ 1
***θFa***	live streaming platform fines the live streaming anchor when the live streaming anchor and the supplier collude, *θFa* ≥ 0
***θFs***	live streaming platform fines the supplier when the live streaming anchor and the supplier collude, *θFs* ≥ 0
***γ***	revenue sharing ratio when the live streaming anchor and the supplier collude, 0 ≤ *γ* ≤ 1
***γDa***	sum received by the live streaming anchor when the live streaming anchor and the supplier collude, *γDa* ≥ 0
***γDs***	sum received by the supplier when the live streaming anchor and the supplier collude, *γDs* ≥ 0

### 3.2. Revenue matrix

According to the above assumptions, the payoff matrix of the game between the live streaming anchor, supplier, and live streaming platform is listed in Table 2.

**Table 2:** Payoff matrix of the game subjects.

Game Subjects		Live streaming Platform	
Live streaming anchor	Supplier	Strict Regulation (*z*)	Negative Regulation (1 − *z*)
**honesty** **(*x*)**	**honesty** **(*y*)**	*Ea* + (1 − *α*)*Ia* + *λRa* − *Ca*	*Ea* − *Ca*
		*Es* + (1 − *β*)*Is* + *λRs* − *Cs*	*Es* − *Cs*
		*Ep* + *αIa + βIs–λRa–λRs* − *Cp*	*Ep*
	**dishonesty** **(1 − *y*)**	*Ea* + (1 − *α*)*Ia + λRa* − *Ca*	*Ea* − *Ca*
		*Es* − *θFs*	*Es*
		*Ep + αIa + θFs–λRa* − *Cp*	*Ep*
**dishonesty** **(1 − *x*)**	**honesty** **(*y*)**	*Ea–θFa*	*Ea*
		*Es* + (1 − *β*)*Is + λRs* − *Cs*	*Es* − *Cs*
		*Ep + βIs + θFa* − *λRs* − *Cp*	*Ep*
	**dishonesty** **(1 − *y*)**	*Ea* + *γDa–θFa* − *Ta*	*Ea* − *Ta*
		*Es + γDs–θFs* − *Ts*	*Es* − *Ts*
		*Ep + θFa + θFs–Cp* − *Tp*	*Ep* − *Tp*

### 3.3. Analysis of the model

#### 3.3.1. Strategic stability analysis of live streaming anchor

Assuming that the expected returns of the live streaming anchor’s, honesty or dishonesty are *V*_11_ and *V*_12_, respectively, and the average expected return is ${\bar{V}}_{1}$, we obtain the following equations:

$$\begin{array}{l} V_{11}=yz(Ea+(1-\alpha )Ia+\lambda Ra-Ca)+y(1-z)(Ea-Ca)+z(1-y)(Ea+(1-\alpha )\\ \;Ia+\lambda Ra-Ca)+(1-y)(1-z)(Ea-Ca) \end{array}$$ (1)

$$V_{12}=yz(Ea-\theta Fa)+y(1-z)Ea+z(1-y)(Ea+\gamma Da-\theta Fa)+(1-y)(1-z)Ea$$ (2)

$${\bar{V}}_{1}=xV_{11}+(1-x)V_{12}$$ (3)

The dynamic replication equation for the choice of the live streaming anchor strategy is as follows:

$$F(x)=\frac{dx}{dt}=x(V_{11}-\bar{V_{1}})=x(1-x)(zIa-Ca-z\alpha Ia-z\gamma Da+z\theta Fa+z\lambda Ra+yz\gamma Da)$$ (4)

The first-order derivative of *F*(*x*) is as follows:

$$\frac{dF(x)}{dx}=(1-2x)(zIa-Ca-z\alpha Ia-z\gamma Da+z\theta Fa+z\lambda Ra+yz\gamma Da)$$ (5)

Then, three situations are discussed based on formula (4):

Clearly, *x* = 0, *x* = 1, and $z=\frac{Ca}{Ia-\alpha Ia-\gamma Da+\theta Fa+\lambda Ra+y\gamma Da}$ are the roots of the *F*(*x*) = 0. According to the stability principle proposed by Friedman (1991), *x* is the evolutionary stable state (ESS) point when *F*(*x*) = 0 and *F*′(*x*) ≤ 0.

1. When $z=\frac{Ca}{Ia-\alpha Ia-\gamma Da+\theta Fa+\lambda Ra+y\gamma Da}$, the strategy is stable for any *x* ∈ [0, 1].
2. When $z=\frac{Ca}{Ia-\alpha Ia-\gamma Da+\theta Fa+\lambda Ra+y\gamma Da}$, ${\left.\frac{dF(x)}{dx}\right|}_{x=0}<0$, and ${\left.\frac{dF(x)}{dx}\right|}_{x=1}>0$, x = 0, the strategy is stable and the live streaming anchor chooses the dishonesty strategy.
3. When $\frac{Ca}{Ia-\alpha Ia-\gamma Da+\theta Fa+\lambda Ra+y\gamma Da}<z\leq 1$, ${\left.\frac{dF(x)}{dx}\right|}_{x=0}>0$, and ${\left.\frac{dF(x)}{dx}\right|}_{x=1}<0$, *x* = 1, the strategy is stable, and the live streaming anchor chooses the honesty strategy.

#### 3.3.2. Strategic stability analysis of suppliers

We assume that the expected benefits of the supplier’s honesty or dishonesty are *V*_21_ and V_22_, respectively, and the average expected benefit is ${\bar{V}}_{2}$. Then, we obtain the following equations:

$$\begin{array}{l} V_{21}=xz(Es+(1-\beta )Is+\lambda Rs-Cs)+x(1-z)(Es-Cs)+(1-x)z(Es+(1-\beta )Is\\ \;+\lambda Rs-Cs)+(1-x)(1-z)(Es\text{-}Cs\text{)} \end{array}$$ (6)

$$V_{22}=xz(Es-\theta Fs)+x(1-z)Es+(1-x)z(Es+\gamma Ds-\theta Fs)+(1-x)(1-z)Es$$ (7)

$${\bar{V}}_{2}=yV_{\text{21}}+(1-y)V_{22}$$ (8)

The dynamic replication equations for supplier strategy selection and the first derivatives are given below:

$$\begin{array}{l} F(y)=\frac{dy}{dt}=y(V_{21}-\bar{V})=y(1-y)(V_{21}-V_{22})\\ =y(1-y)(zIs-Cs-z\gamma Ds-z\beta Is+z\theta Fs+z\lambda Rs+xz\gamma Ds) \end{array}$$ (9)

$$\frac{dF(y)}{dy}=(1-2y)(zIs-Cs-z\gamma Ds-z\beta Is+z\theta Fs+z\lambda Rs+xz\gamma Ds)$$ (10)

Then, three situations are discussed based on formula (9):

Clearly, *y* = 0, *y* = 1, and $z=\frac{Cs}{Is-\gamma Ds-\beta Is+\theta Fs+\lambda Rs+x\gamma Ds}$ are the roots of *F*(*y*) = 0. According to the stability principle proposed by Friedman (1991), *y* is the ESS point when

*F*(*y*) = 0 and *F*′(*y*) ≤ 0.

1. When $z=\frac{Cs}{Is-\gamma Ds-\beta Is+\theta Fs+\lambda Rs+x\gamma Ds}$, the strategy is stable for any *y* ∈ [0, 1].
2. When $0\leq z\leq \frac{Cs}{Is-\gamma Ds-\beta Is+\theta Fs+\lambda Rs+x\gamma Ds}$, ${\left.\frac{dF(y)}{dy}\right|}_{y=0}<0$, and ${\left.\frac{dF(y)}{dy}\right|}_{y=1}>0$, *y* = 0, the strategy is evolutionarily stable, and the supplier chooses the dishonesty strategy.
3. When $\frac{Cs}{Is-\gamma Ds-\beta Is+\theta Fs+\lambda Rs+x\gamma Ds}<z\leq 1$, ${\left.\frac{dF(y)}{dy}\right|}_{y=0}>0$, and ${\left.\frac{dF(y)}{dy}\right|}_{y=1}<0$, *y* = 1, the strategy is stable, and the supplier chooses the honesty strategy.

#### 3.3.3. Strategic stability analysis of live streaming platform

Assuming that the expected benefits of supplier honesty or dishonesty are *V*_31_ and *V*_32_, respectively, and the average expected benefit is ${\bar{V}}_{3}$, we get the following:

$$\begin{array}{l} V_{31}=xy(Ep+\alpha Ia+\beta Is-\lambda Ra-\lambda Rs-Cp)+x(1-y)(Ep+\alpha Ia+\theta Fs-\lambda Ra-Cp)\\ +y(1-x)(Ep+\beta Is+\theta Fa-\lambda Rs-Cp)+(1-x)(1-y)(Ep+\theta Fa+\theta Fs-Cp) \end{array}$$ (11)

$$V_{32}=xyEp+x(1-y)Ep+y(1-x)Ep+(1-x)(1-y)Ep$$ (12)

$${\bar{V}}_{3}=zV_{31}+(1-z)V_{32}$$ (13)

The dynamic replication equations of live streaming platform strategy selection and first-order derivatives are as follows:

$$\begin{array}{l} F(\text{z})=\frac{dz}{dt}=z\left(V_{31}-{\bar{V}}_{3}\right)=z(1-z)\left(V_{31}-V_{32}\right)\\ =z(z-1)(Cp-\theta Fa-\theta Fs-x\alpha Ia-y\beta Is+x\theta Fa+y\theta Fs+x\lambda Ra+y\lambda Rs\text{)} \end{array}$$ (14)

$$\frac{dF(z)}{dz}=(2z-1)(Cp-\theta Fa-\theta Fs-x\alpha Ia-y\beta Is+x\theta Fa+y\theta Fs+x\lambda Ra+y\lambda Rs)$$ (15)

Then, three situations are discussed based on formula (14):

Clearly, *z* = 0, *z* = 1, and $y=\frac{\theta Fa+\theta Fs+x\alpha Ia-Cp-x\theta Fa-x\lambda Ra}{\theta Fs+\lambda Rs-\beta Is}$ are the roots of *F*(*z*) = 0. According to the stability principle by Friedman (1991), y is the ESS point when *F*(*z*) = 0 and *F*′(*z*) ≤ 0.

1. When $y=\frac{\theta Fa+\theta Fs+x\alpha Ia-Cp-x\theta Fa-x\lambda Ra}{\theta Fs+\lambda Rs-\beta Is}$, the strategy is stable for any *z* ∈ [0, 1].
2. When $0\leq y\leq \frac{\theta Fa+\theta Fs+x\alpha Ia-Cp-x\theta Fa-x\lambda Ra}{\theta Fs+\lambda Rs-\beta Is}$, ${\left.\frac{dF(z)}{dz}\right|}_{z=0}<0$, and ${\left.\frac{dF(z)}{dz}\right|}_{z=1}>0$, *z* = 0, the strategy is evolutionarily stable. and the live streaming platform chooses a negative regulatory strategy.
3. When $\frac{\theta Fa+\theta Fs+x\alpha Ia-Cp-x\theta Fa-x\lambda Ra}{\theta Fs+\lambda Rs-\beta Is}<y\leq 1$, ${\left.\frac{dF(z)}{dz}\right|}_{z=0}>0$, and ${\left.\frac{dF(z)}{dz}\right|}_{z=1}<0$, *z* = 1, the strategy is evolutionarily stable, and the platform chooses a proactive regulatory strategy.

#### 3.3.4. Stability analysis of model

This section constructs and solves the dynamic replication equations of the three-party game among the live streaming anchor, supplier, and live streaming platform to demonstrate the circumstances and methodology of the stable strategy formation of the evolutionary game in the live streaming.

Multiple sets of possible solutions were obtained from *F*(*x*) = 0, *F*(*y*) = 0, and *F*(*z*) = 0. As the stable solutions in multiple-group evolutionary games are strict Nash equilibria, Lyapunov’s first law is used for stability analysis for only eight pure strategic equilibria in the evolutionary system *E*_1_ (0,0,0), *E*_2_ (1,0,0), *E*_3_ (0,1,0), *E*_4_ (0,0,1), *E*_5_ (1,1,0), *E*_6_ (1,0,1), *E*_7_ (0,1,1), and *E*_8_ (1,1,1).

According to Friedman’s method, the stability of the equilibrium point of the differential system can be achieved by analyzing the eigenvalues of the Jacobi matrix of the system as follows:

$$J=\left(\begin{array}{ccc} \frac{\partial F(x)}{\partial x} & \frac{\partial F(x)}{\partial y} & \frac{\partial F(x)}{\partial z}\\ \frac{\partial F(y)}{\partial x} & \frac{\partial F(y)}{\partial y} & \frac{\partial F(y)}{\partial z}\\ \frac{\partial F(z)}{\partial x} & \frac{\partial F(z)}{\partial y} & \frac{\partial F(z)}{\partial z} \end{array}\right)$$ (16)

Following Lyapunov’s first method [47], when all eigenvalues are negative, the equilibrium is a stable point; when the eigenvalues are all positive, the equilibrium is an unstable point; and when the eigenvalues are positive and negative, a saddle point is reached. The eigenvalues and stabilities of each point are presented in Tables 3 and 4, respectively.

**Table 3:** Eigenvalues of the Jacobian matrix for equilibrium points.

Balancing Point	Eigenvalue 1	Eigenvalue 2	Eigenvalue 3
***E***_**1**_ **(0,0,0)**	− *Ca*	− *Cs*	*θFa* − *Cp + θFs*
***E***_**2**_ **(0,0,1)**	*Ia* − *Ca* − *αIa* − *γDa + θFa + λRa*	*Is* − *Cs* − *γDs* − *βIs + θFs + λRs*	*Cp* − *θFa* − *θFs*
***E***_**3**_ **(0,1,0)**	*− Ca*	*Cs*	*βIs* − *Cp + θFa* − *λRs*
***E***_**4**_ **(1,0,0)**	*Ca*	*− Cs*	*αIa*− *Cp + θFs* − *λRa*
***E***_**5**_ **(0,1,1)**	*Ia* − *Ca* − *αIa + θFa + λRa*	*Cs* − *Is + γDs + βIs* − *θFs* − *λRs*	*Cp* − *βIs* − *θFa + λRs*
***E***_**6**_ **(1,1,0)**	*Ca*	*Cs*	*αIa* − *Cp + βIs* − *λRa* − *λRs*
***E***_**7**_ **(1,0,1)**	*Ca* − *Ia + αIa + γDa* − *θFa* − *λRa*	*Is* − *Cs* − *βIs + θFs + λRs*	*Cp* − *αIa* − *θFs + λRa*
***E***_**8**_ **(1,1,1)**	*Ca* − *Ia + αIa* − *θFa* − *λRa*	*Cs* − *Is + βIs* − *θFs* − *λRs*	*Cp* − *αIa* − *βIs + λRa + λRs*

**Table 4:** Stability analysis of equilibrium points.

Equilibrium Points	Stability Conditions
***E***_**1**_ **(0,0,0)**	*θFa + θFs < Cp*
***E***_**2**_ **(0,0,1)**	*Ia + θFa + λRa < Ca + αIa + γDa*, *Is + θFs + λRs < Cs + γDs + βIs*, *Cp < θFa + θFs*
***E***_**3**_ **(0,1,0)**	unstable point
***E***_**4**_ **(1,0,0)**	unstable point
***E***_**5**_ **(0,1,1)**	*Ia + θFa + λRa < Ca + αIa*, *Cs + γDs + βIs < Is + θFs + λRs*, *Cp + λRs < βIs + θFa*
***E***_**6**_ **(1,1,0)**	unstable point
***E***_**7**_ **(1,0,1)**	*Ca + αIa + γDa < Ia + θFa + λRa*, *Is + θFs + λRs < Cs + βIs*, *Cp + λRa < αIa + θFs*
***E***_**8**_ **(1,1,1)**	*Ca + αIa < Ia + θFa + λRa*, *Cs + βIs < Is + θFs + λRs*, *Cp + λRa +λRs < αIa + βIs*

Combining the settings of the parameter sizes in the model shows that *Ca* > 0 and *Cs* > 0; therefore, only *E*_1_ (0,0,0), *E*_2_ (1,0,0), *E*_5_ (1,1,0), *E*_7_ (0,1,1), and *E*_8_ (1,1,1) present for the five cases of evolutionary stabilization strategies.

**Scenario 1:** Expressed by the eigenvalue of stability point *E*_1_. The three-party evolutionary stabilization strategy is {dishonesty, dishonesty, negative regulation}. The severity of the punishment is the key to selecting the live streaming platform. If the punishment cannot offset the cost of strict regulation of the live streaming platform, then the live streaming platform will choose the negative regulation strategy. In this Scenario, the live streaming anchor and supplier will not face fines when they adopt collusion strategy. Speculation also contributes toward the live streaming anchor and supplier violating policies, and the system shows value co-destruction.
**Scenario 2:** Expressed by the *E*_2_ eigenvalue expression. The limitations affecting the strategy of the live streaming platform are opposed to the constraints in Scenario 1. The live streaming platform tends to strict regulate when the expected gains of strict regulation exceed the costs of strict regulation. In this Scenario, the live streaming anchor and supplier need to measure the loss caused by the live streaming platform penalties when they violate the law. Suppose that the total amount of network externality benefits and incentives that the honesty strategy can obtain is less than the difference between the benefits and fines when the collusion strategy evolves to stabilize the strategy as {dishonesty, dishonesty, strict regulation}. In this case, the violations by the live streaming anchor and supplier, and the mismatch between the strict regulation strategy and the live streaming platform will trigger value co-destruction.
**Scenario 3:** Expressed by the *E*_5_ eigenvalue expression. In Scenario 3, the live streaming platform chooses a strict regulation strategy when the anticipated gains of strict regulation are greater than the costs of strict regulation. The variables affecting the supplier’s strategy choice are the same as those in Scenario 2. However, the critical conditions are reversed: the supplier changes its strategy to adopt a positive one. In this Scenario, when the benefit (to the live streaming anchor) of choosing an illegal strategy is greater than the cost of honesty, the live streaming anchor tends to adopt a collusion strategy, and the system evolves into a stable strategy of {dishonesty, honesty, strict regulation}.
**Scenario 4:** Expressed by the *E*_7_ eigenvalue expression. In Scenario 4, the variables affecting the strategy selection of the live streaming anchor and supplier do not change compared to Scenario 2. However, the critical conditions are reversed when the live streaming anchor tends to perform negatively, the supplier chooses honest behavior, and the live streaming platform takes punitive measures for the live streaming anchor and incentivizes the supplier by sharing network externality benefits with the supplier. The system tends to {honesty, dishonesty, strict regulation}, and resources abuse by the live streaming anchor or the supplier reduces the overall value of the system if the live streaming platform does not meet the expectations of the live streaming anchor and supplier due to docking dysfunction triggered by value co-destruction.
**Scenario 5:** Expressed by the *E*_8_ eigenvalue expression. When the cost of strict regulation of the live streaming platform is less than the gain from network externality, and the difference between the cost of the positive strategy and extra gain of the live streaming anchor and supplier is less than the loss of the penalty imposed by the negative strategy, {honesty, honesty, strict regulation} is the system evolutionary stability strategy under the condition that all three parties make reasonable use of resources. All can buttress the other parties’ performance with positive behavior. Value co-destruction transforms into value co-creation. Generally, when the general ecosystem of the industry is more relaxed, the live streaming platform tends not to take a higher penalty for the rapid accumulation of traffic and the fastest speed at reaching the minimum network effect; that is, *θ* accepts a smaller value. However, as the industry and the scales of its associated live streaming platforms expand, the violations become more frequent. In this Scenario, the live streaming anchors and suppliers receive more penalties for violations, so they are incentivized to be honest. Overall, the live streaming platform that provides appropriate incentives and punishments is the key to achieving ESS. This Scenario is the ideal state and desired ESS point.

## 4. Simulation and analysis of evolutionary game models

### 4.1. Basic simulation analysis

**Fig 2:**
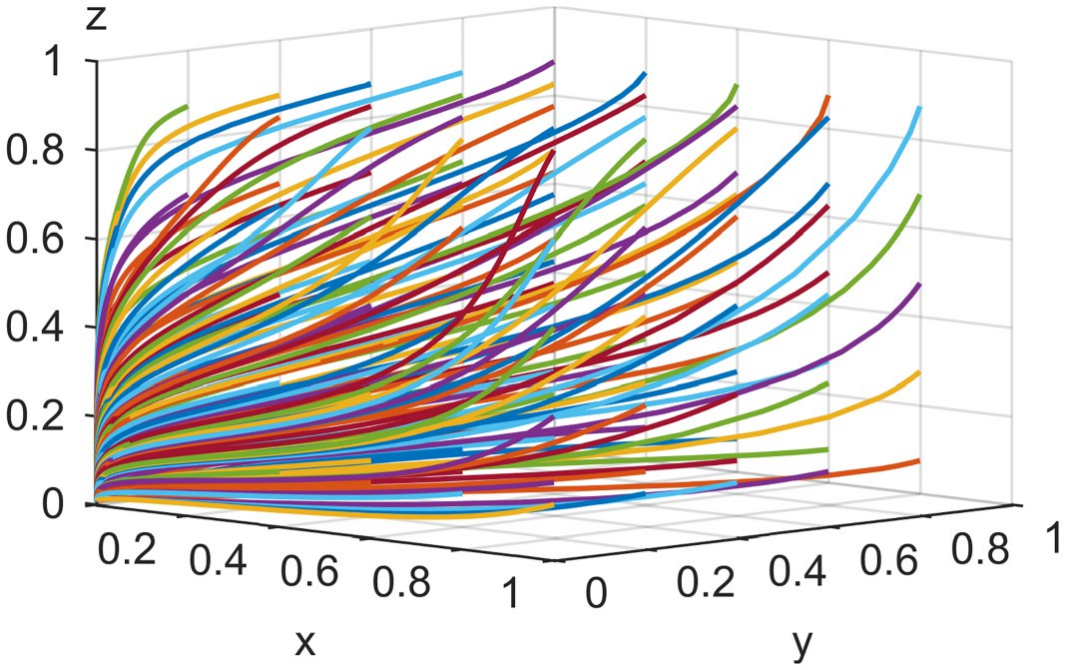
Basic simulation results for scenario 1.

**Fig 3:**
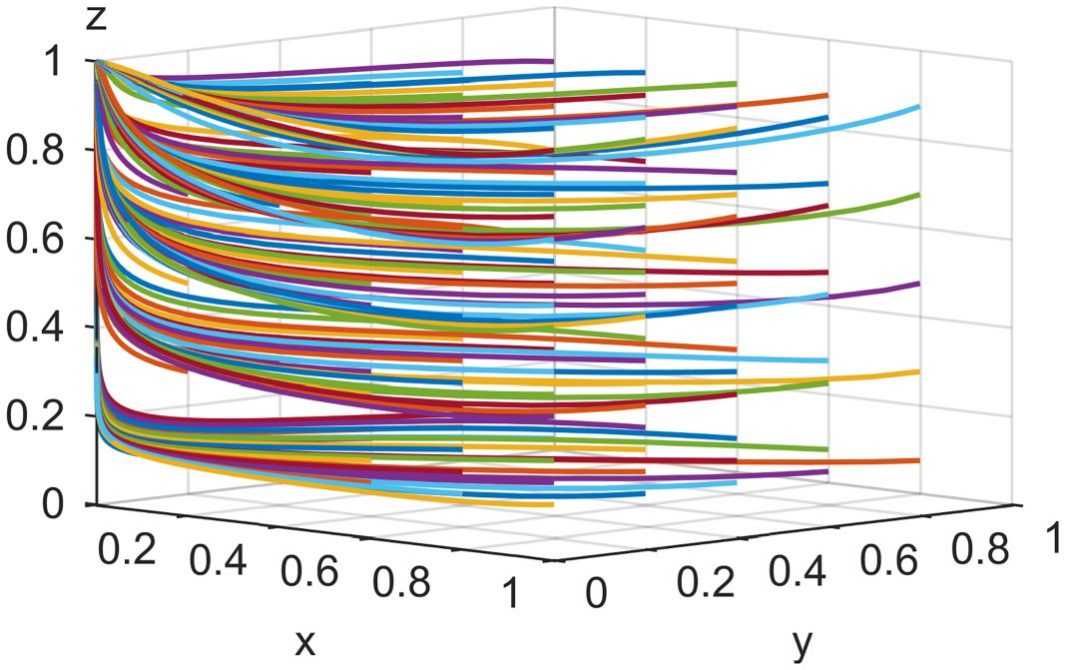
Basic simulation results for scenario 2.

**Fig 4:**
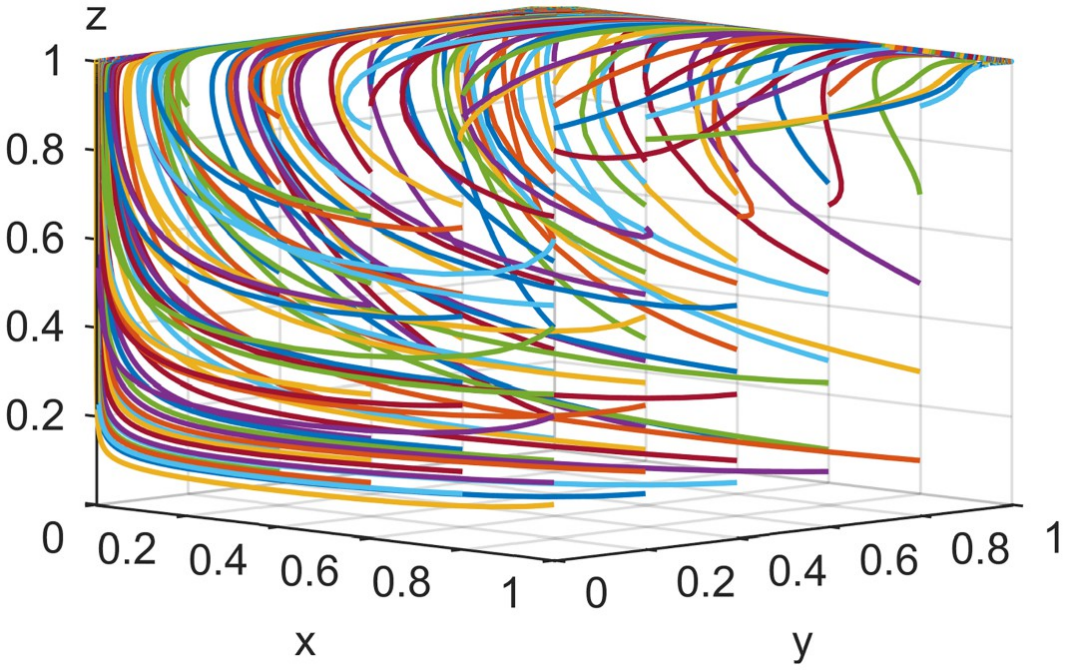
Basic simulation results for scenario 3.

**Fig 5:**
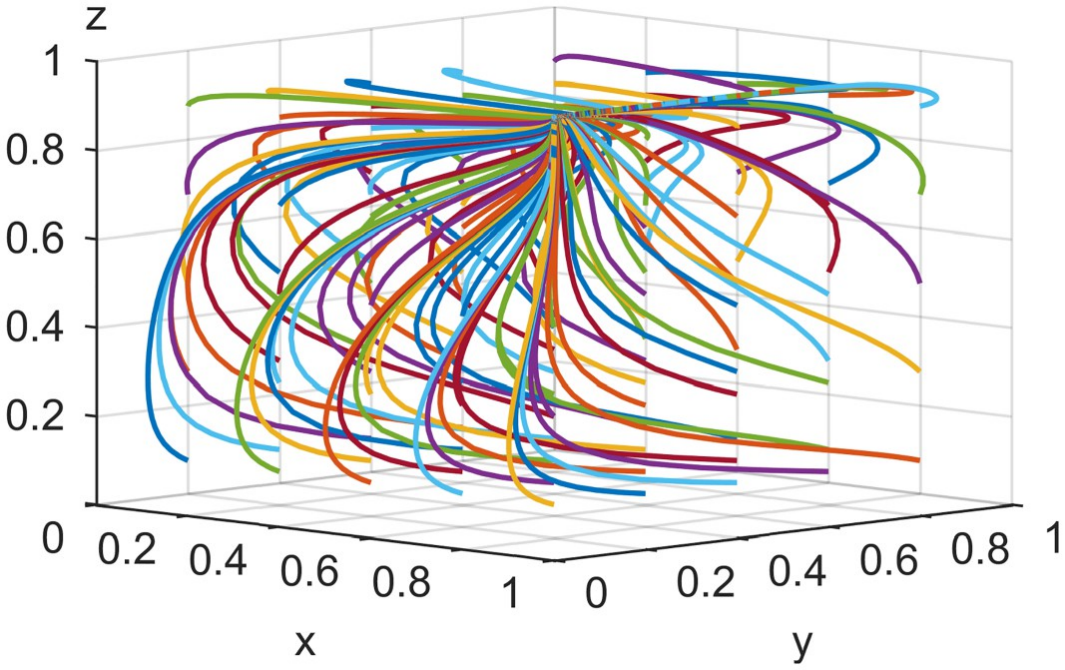
Basic simulation results for scenario 4.

**Fig 6:**
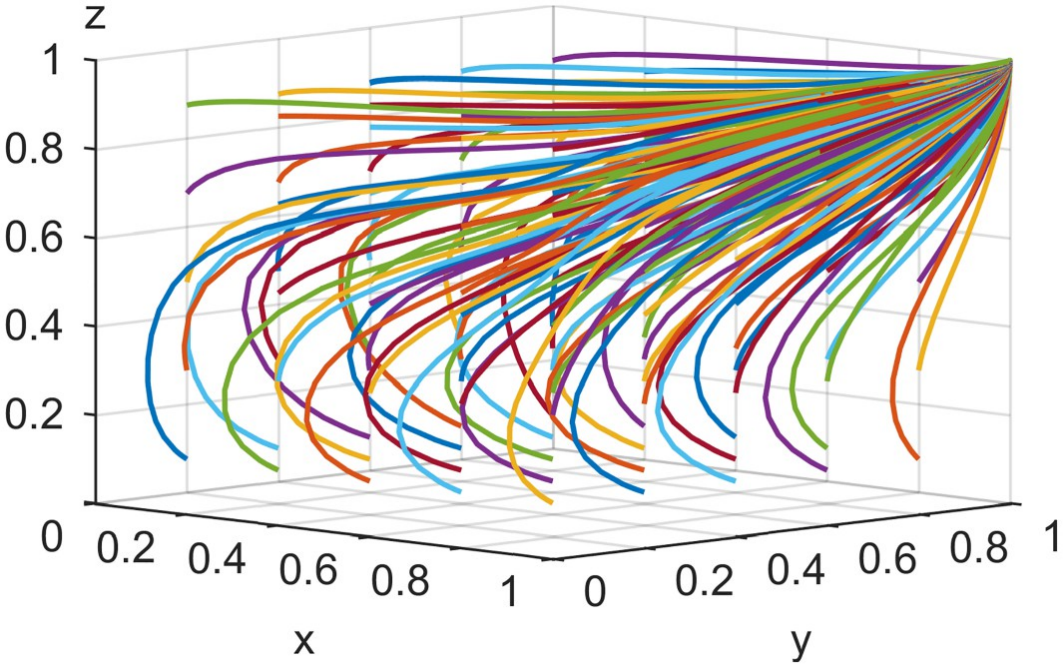
Basic simulation results for scenario 5.

To check the validity of the above model analysis results, the parameters were assigned according to the above settings and constraints in different cases based on [46]. For Scenario 1, *Ea* = 2, *Es* = 6, *Ep* = 1, *Ca* = 5, *Cs* = 5, *Cp* = 6, *Ia* = 5, *Is* = 4, α = 0.5, *β* = 0.5, *Ra* = 3, *Rs* = 1, *λ* = 0.3, *γ* = 0.5, *Fa* = 6, *Fs* = 4, *θ* = 0.5, *Da* = 2, *Ds* = 6; for Scenario 2, *Ca* = 6, *Cp* = 4, other parameters are the same as in Scenario 1; for Scenario 3, *Ca* = 5, *Cs* = 3, *Ia* = 3, *Is* = 8, other parameters are the same as in Scenario 2; for in Scenario 4, *Ca* = 1, *Cs* = 5, *Cp* = 2, *Ia* = 5, *Is* = 4; other parameters are the same as in Scenario 3; for Scenario 5, *Cs* = 1, *Cp* = 1, *Fa* = 4, *Fs* = 2, other parameters are the same as in Scenario 4. MATLAB simulated the values of the five Scenarios, and Figs 2–6 were obtained. The validity of the theoretical analysis was verified.

### 4.2. Initial strategy simulation analysis

**Fig 7:**
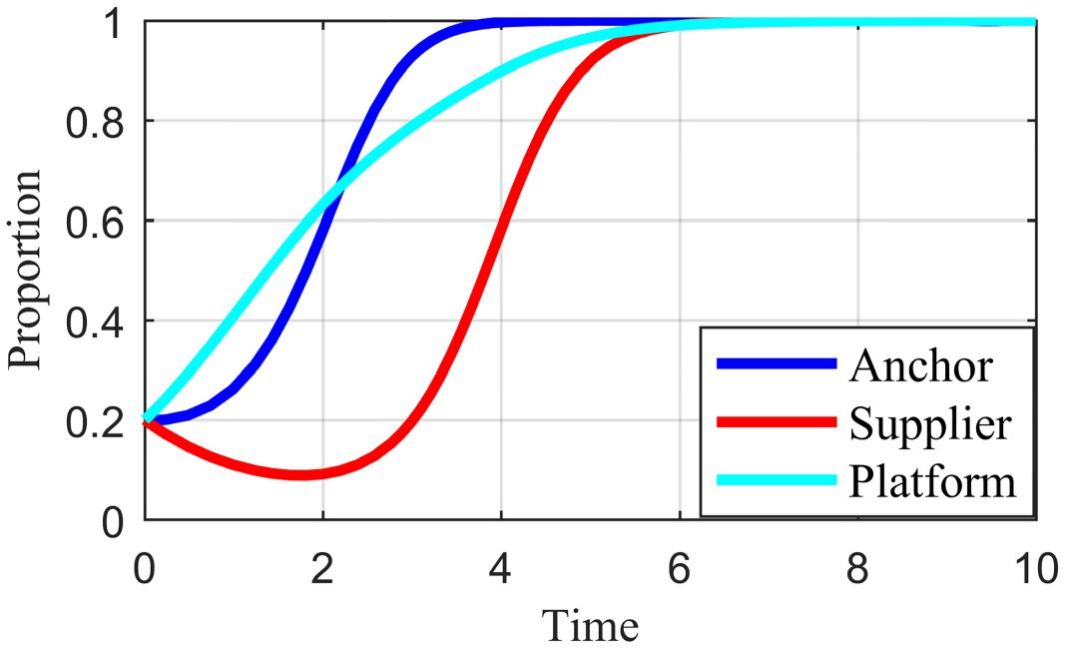
Effect of initial values on evolutionary results (x = y = z = 0.2).

**Fig 8:**
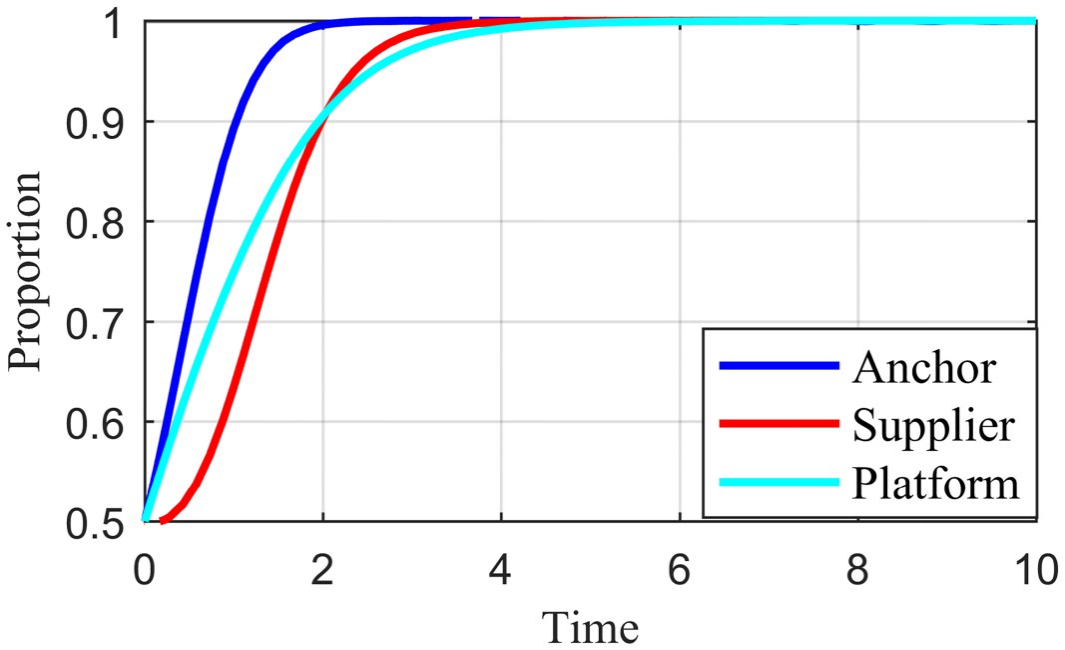
Effect of initial values on evolutionary results (x = y = z = 0.5).

**Fig 9:**
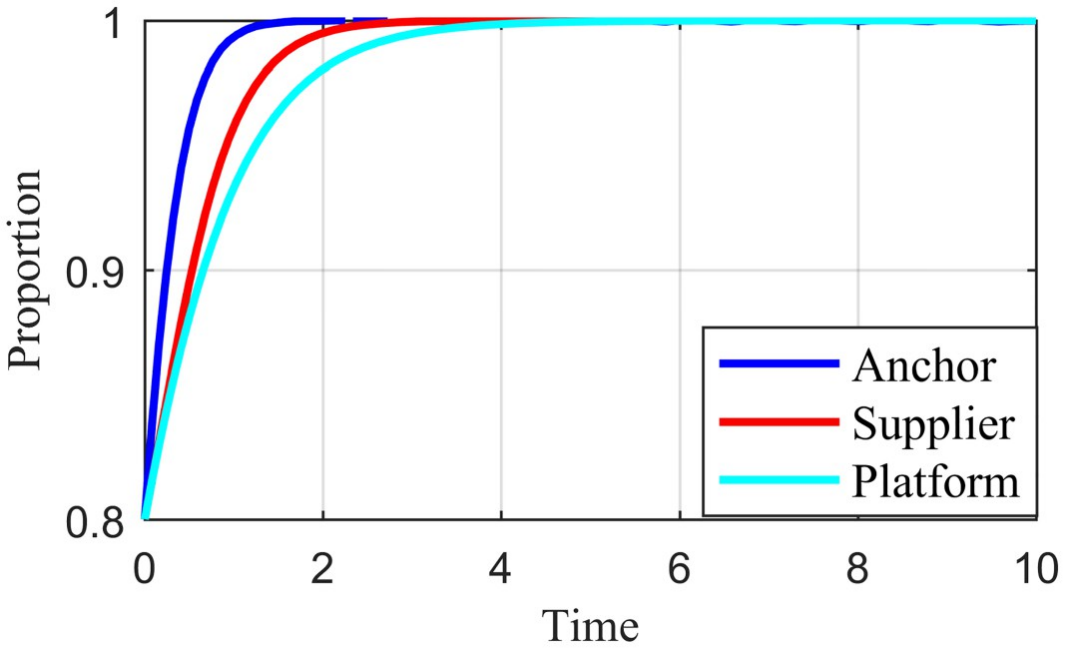
Effect of initial values on evolutionary results (x = y = z = 0.8).

When the live streaming anchor and supplier operate in good faith, and the live streaming platform is strictly regulated, then this situation is good for the sustainable development of the live streaming industry; that is, *E*_*8*_ (1,1,1) in Scenario 5 is the ideal ESS point. Based on the values assigned to each parameter in Scenario 5 in Section 4.1, it is assumed that the initial values of the strategy selection of the live streaming anchor, supplier, and the live streaming platform are as follows: *x* = 0.2, *y* = 0.2, *z* = 0.2; *x* = 0.5, *y* = 0.5, *z* = 0.5; *x* = 0.8, *y* = 0.8, *z* = 0.8. The experimental results of the numerical simulations for different initial values are shown in Figs 7–9.

As shown in Figs 7–9, the different initial values of the three subjects (live streaming anchor, supplier, and live streaming platform) have no impact on the ultimate stable state, which eventually stabilizes at point *E*_5_ (1,1,1). However, a change in the initial values affects the time the system reaches a steady state.

Using the results in Fig 7, for example, when the primary probability of the three subjects is relatively low (e.g., *x* = *y* = *z* = 0.2), the suppliers may experience instability in their behavioral strategy at the early stage of development because of speculative psychology. This instability is influenced by the strict regulation of the live streaming platform and the honest strategy of the anchor, making the suppliers finally choose an honest strategy. Finally, the system evolves to point *E*_5_ (1,1,1). Figs 8 and 9 show that as the initial probability increases, the live streaming anchor and the supplier gradually give up their dishonesty strategy choices, and the system evolves toward the *E*_5_ (1,1,1) point. We also find that the time for the system to reach stability becomes shorter as the initial probability of the tripartite subject keeps increasing. For example, Fig 9 shows the shortest time to reach the steady state, and Fig 7 shows the longest time. This indicates that when live streaming platforms choose to regulate strictly, the live streaming anchors and suppliers will make honest behavioral strategy choices more quickly.

### 4.3. Parameter sensitivity analysis

#### 4.3.1. Analysis of the simulation results on the impact of incentive strength

**Fig 10:**
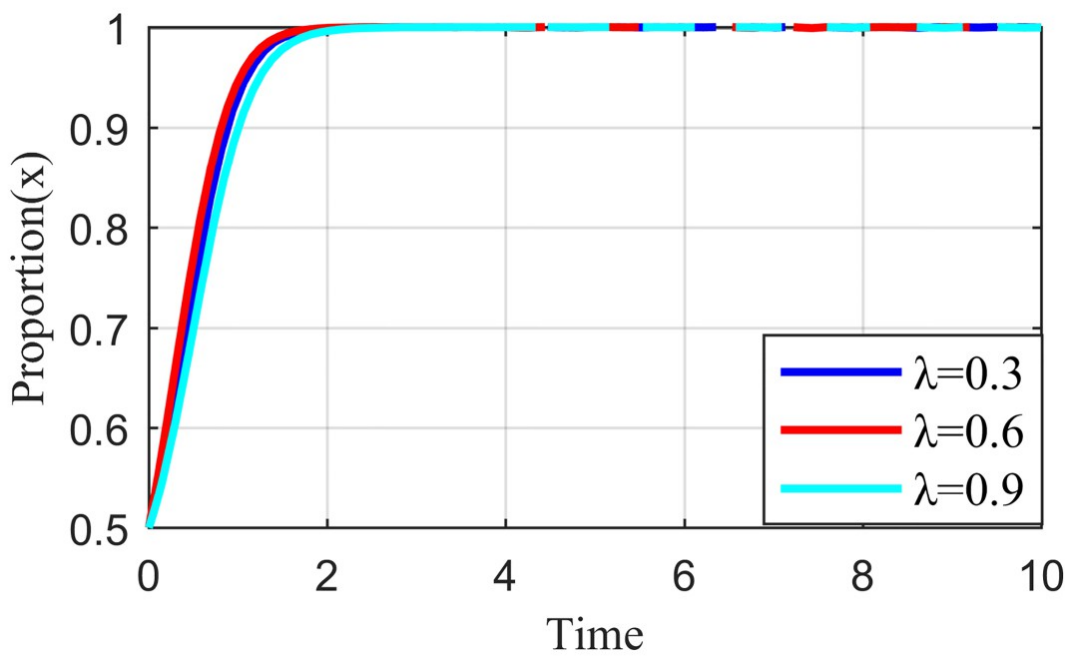
Effect of reward intensity on live streaming anchor.

**Fig 11:**
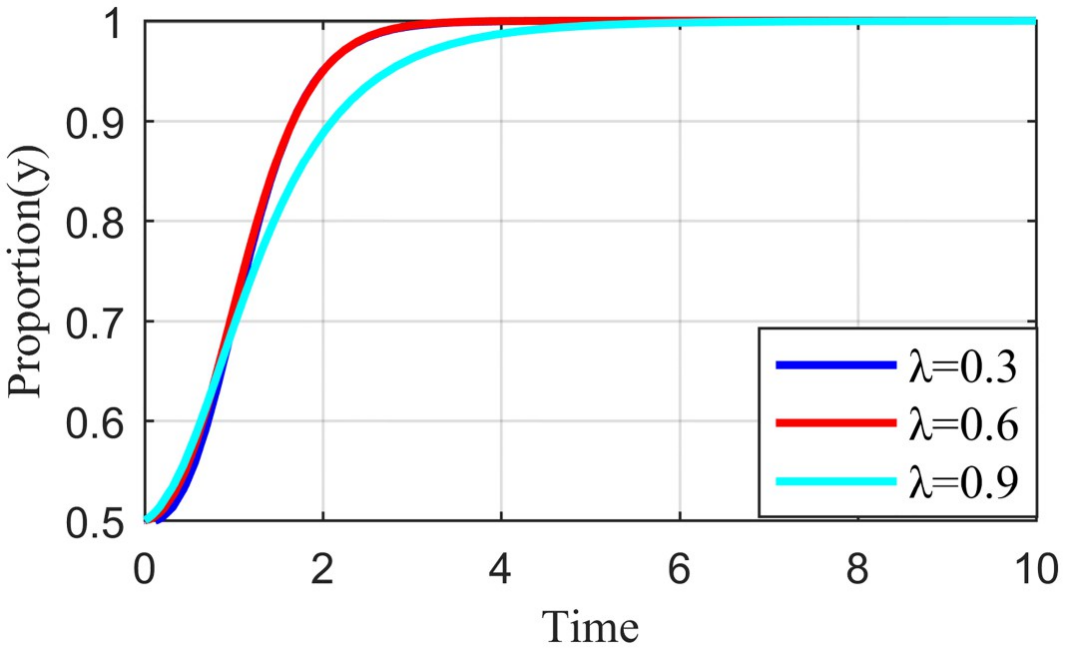
Effect of reward intensity on supplier.

**Fig 12:**
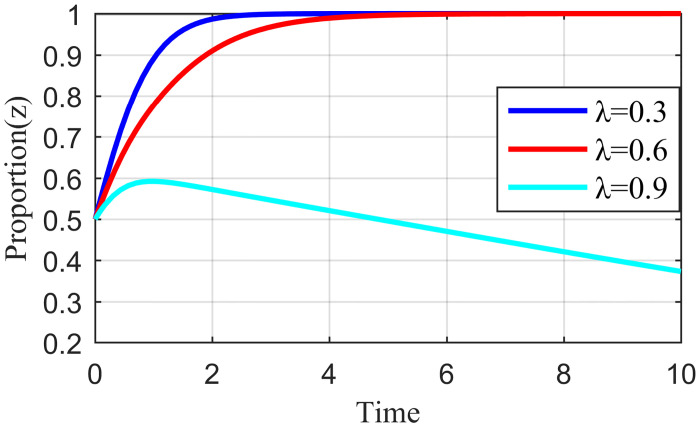
Effect of reward intensity on live streaming platform.

Incentive strength refers to the strict supervision of the live streaming platform under the honesty of the live streaming anchor and supplier strategy to apply specific incentives so that the live streaming anchor and supplier can continue this positive strategic tendency. These incentives include traffic recommendations, official training, and live streaming platform subsidies. Fixing the other parameters at constant values, we let the value of *λ* be equal to 0.3, 0.6, and 0.9, respectively. The simulation results are shown in Figs 10–12.

As shown in Figs 10 and 11, the change in reward intensity *λ* does not substantially influence the evolutionary trajectories of the live streaming anchor and supplier’s behavioral strategies. Eventually, the live streaming anchor and supplier will choose the behavioral honesty strategy, and compared with the supplier, the live streaming anchor will be the first to make ethical behavioral decisions. When *λ* is 0.3 or 0.6, the time taken by the live streaming anchor and supplier to converge on the behavioral strategy of honesty is the same, whereas when *λ* = 0.9, the time to reach stability becomes longer for the live streaming anchor and supplier, which may be influenced by the strategy choice of live streaming platform. Fig 12 shows that the behavioral strategy evolution trend of live streaming platform is different when the reward intensity is different, and when the reward intensity *λ* = 0.3 or *λ* = 0.6, the live streaming platform will eventually choose strict supervision. The time required for *λ* = 0.3 to stabilize is shorter than that required for *λ* = 0.6. When *λ* = 0.9, the live streaming platform will increase its cost due to excessive reward strength, and its strategy will oscillate. It cannot reach a stable state, which will affect the value co-creation and thus affect the benign and sustainable development of the live streaming industry. Therefore, the critical interval of the reward strength *λ* lies between [0.3, 0.6]. Higher incentives can have a negative impact on live streaming platform revenue. However, live streaming platforms need to be brave enough to shoulder social responsibility and balance their own earnings with social responsibility.

#### 4.3.2. Analysis of the simulation results on the impact of punishment intensity

**Fig 13:**
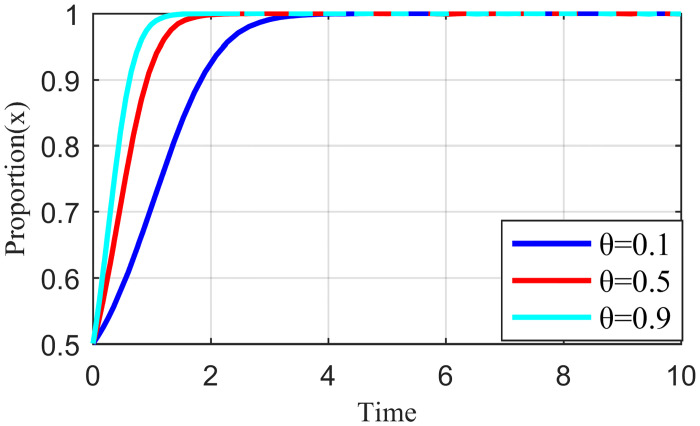
Effect of penalty intensity on live streaming anchor.

**Fig 14:**
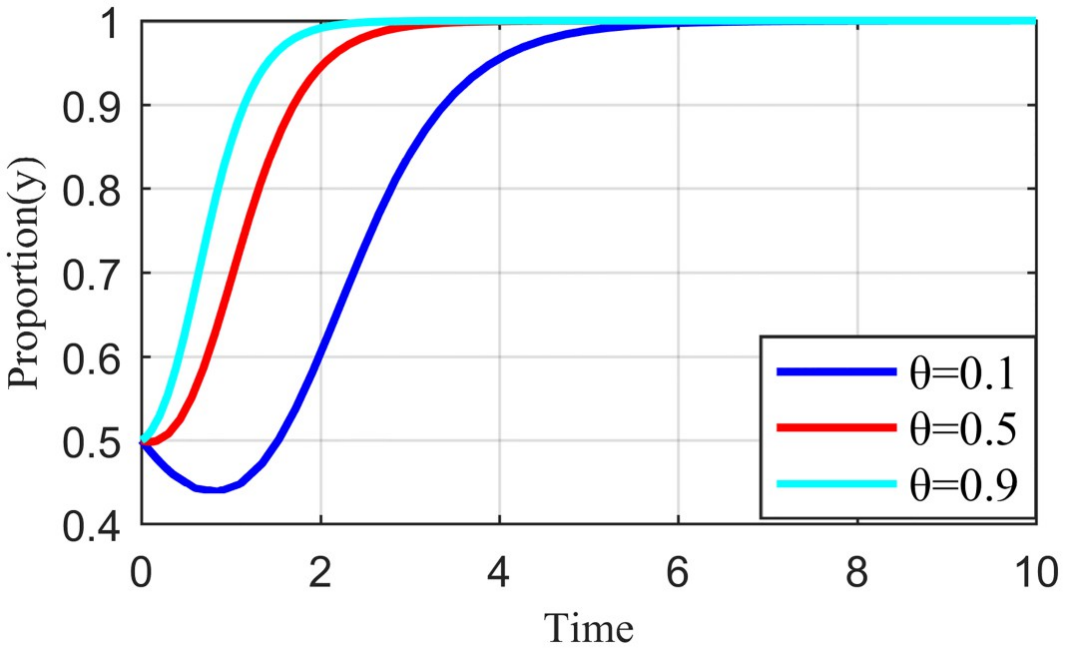
Effect of penalty intensity on supplier.

**Fig 15:**
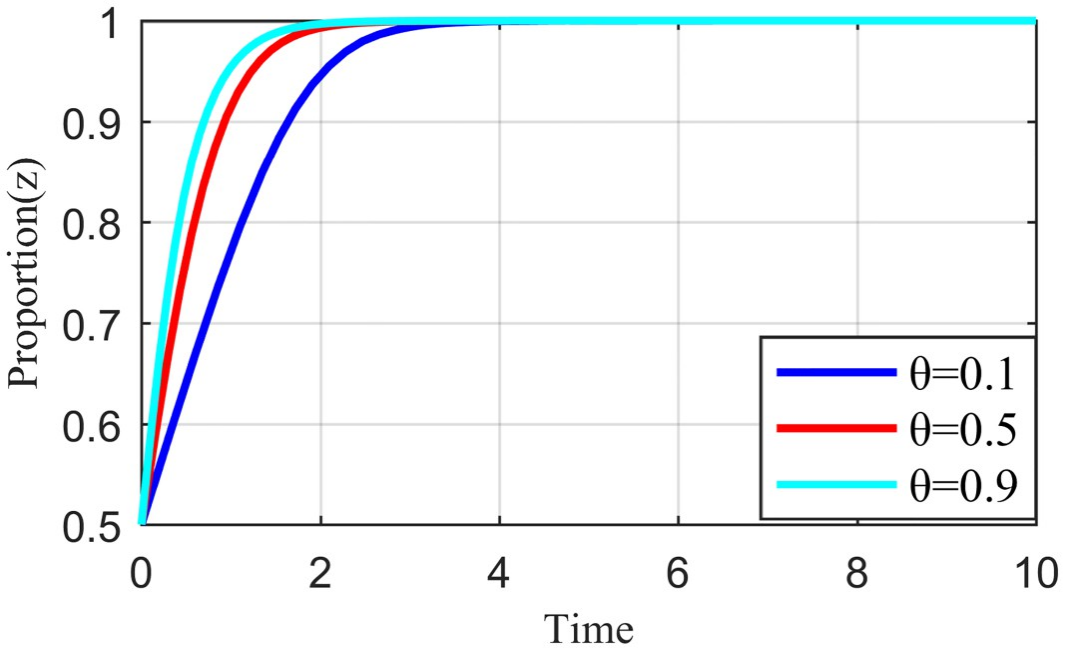
Effect of penalty intensity on live streaming platform.

Penalty strength refers to a certain degree of punitive measures adopted by the live streaming platform for the illegal collusion of the anchor and supplier, such as limiting the flow of suspension, imposing fines, and blocking the number. If the punishment is not sufficiently strong, the violation cost of the live streaming anchor and the supplier will be significantly reduced, which may increase the probability of the live streaming anchor and supplier participating in collusion. To study the influence of the penalty intensity *θ* on the outcome of the three-party evolution game, it is assumed that the values of penalty intensity *θ* are 0.1, 0.5, and 0.9 under the condition that other parameters are specified. The results are shown in Figs 13–15.

Figs 13–15 show that with an increase in *θ* in the punishment of speculative behavior by the live streaming platform, the evolution rate of all three subjects (live streaming anchor, supplier, and live streaming platform) accelerates and the time to reach stability becomes shorter. From Fig 14, when *θ* = 0.1, the live streaming platform is less punitive for violations, suppliers will initially have a fluke mentality, and their preliminary choices will tend to be dishonest strategies. If the live streaming platform does not take timely guidance measures at this time, then suppliers’ violations will have a negative demonstration effect. If the live streaming platform and live streaming anchor can guide the supplier to establish the correct values at this time, then the supplier will eventually choose the honesty behavior strategy after evolution. However, it will take longer to reach stability, and there may be a choice of violation strategy in the middle. Figs 13–15 show that the live streaming anchor and live streaming platform take a shorter time to stabilize than the supplier, so it can be concluded that the supplier is relatively sensitive to the penalty intensity θ. The simulation results show that the penalty needs to be moderate. Too little penalty increases the probability of value co-destruction risk. However, increasing the penalty level has a limited effect on the governance of value co-creation. Penalty that is too high will reduce the efficiency of live streaming platform governance, which is not beneficial for the live streaming ecosystem.

#### 4.3.3. Analysis of the simulation results on the impact of network externality benefit distribution

**Fig 16:**
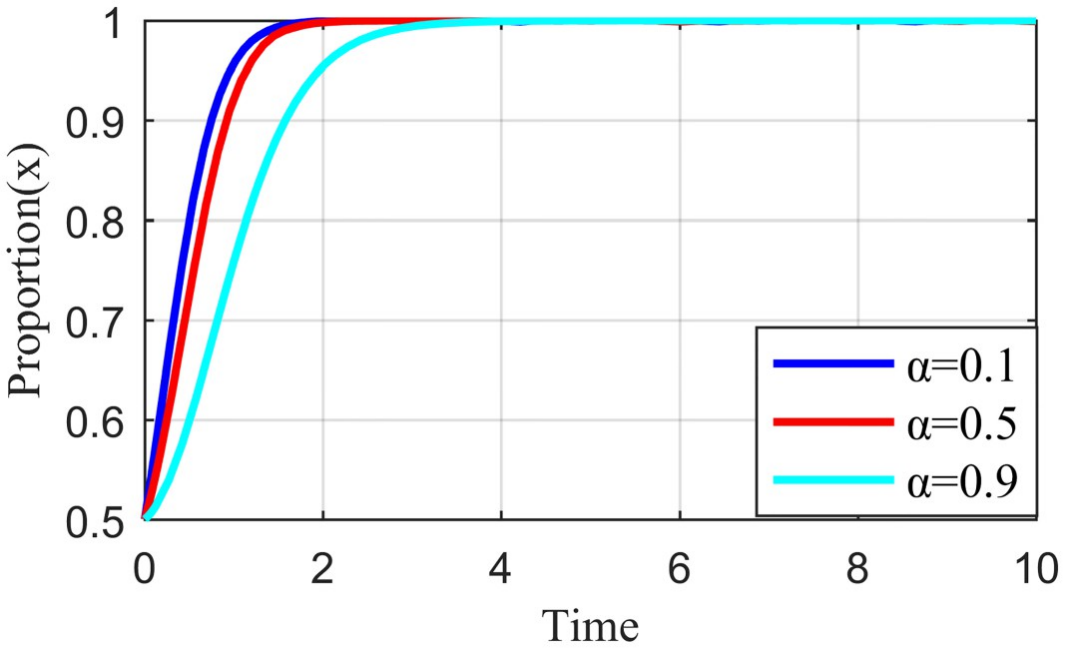
Effect of the revenue allocation coefficient α on live streaming anchor.

**Fig 17:**
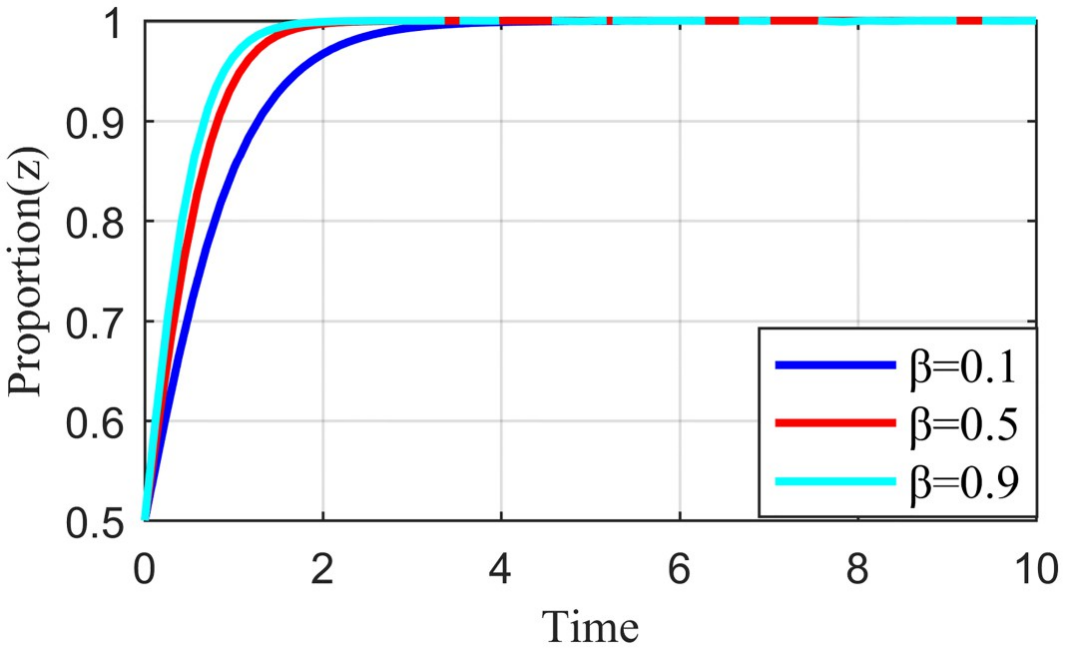
Effect of the revenue allocation coefficient β on live streaming platform.

Network externality benefit refers to the benefit spillover effect shared between the live streaming platform and the live streaming anchor, the live streaming platform and supplier when the live streaming platform has strict regulations, and the live streaming anchor performs their responsibility, or the supplier actively participates in governance. Each subject could change the size of the benefit distribution coefficient through active strategies. For example, when the live streaming platform has less manual intervention and traffic regulation, fans have higher loyalty to the live streaming anchors. This strong social engagement from private domain traffic gives live streaming anchors more space to play. The high and low involvement of the live streaming platform in the traffic distribution mechanism makes α larger and smaller, respectively. Similarly, the level of involvement of the live streaming platform in the evaluation mechanism of suppliers changes the size of *β* accordingly. Keeping the other parameters constant and changing the values of α and *β* so that the values of α are 0.1, 0.5, 0.9, and *β* are 0.1, 0.5, and 0.9, the results are shown in Figs 16 and 17.

**Fig 18:**
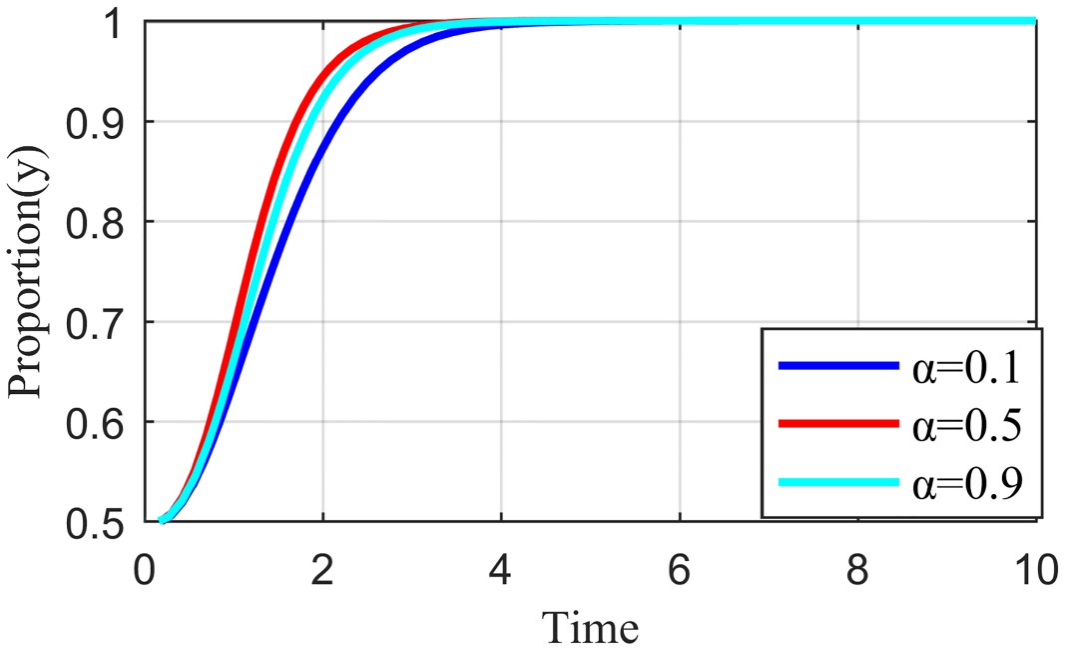
Effect of the revenue allocation coefficient α on supplier.

**Fig 19:**
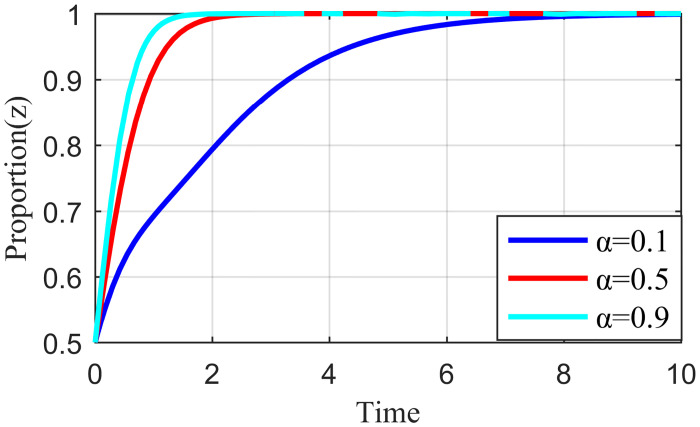
Effect of the revenue allocation coefficient α on live streaming platform.

Fig 16 shows that when α = 0.1, the live streaming anchor’s external revenue allocation weight is significant, and the live streaming anchor that obtains high revenue will quickly choose the honesty behavior strategy. Moreover, as α increases, the live streaming anchor’s external revenue allocation weight gradually decreases, the revenue obtained decreases, and the time required for the live streaming anchor to choose the honesty behavior strategy increases. Fig 18 shows that the strategy choice of suppliers is less influenced by α; when α = 0.5, the time required for suppliers to tend to honesty behavior is the shortest. Fig 19 shows that when α = 0.1, the network externality benefit allocation coefficient of the live streaming platform is smaller and the live streaming platform’s network externality gain is negligible. Moreover, the live streaming platform may pursue an increase in user scale and rise in traffic before deciding its behavioral strategy. The live streaming platform tends to stabilize, and the strategy takes the longest to evolve. When α = 0.9, the network externality gain of the live streaming platform is the largest, the live streaming platform tends to regulate strictly, and the time for the stabilization strategy to evolve is the shortest.

**Fig 20:**
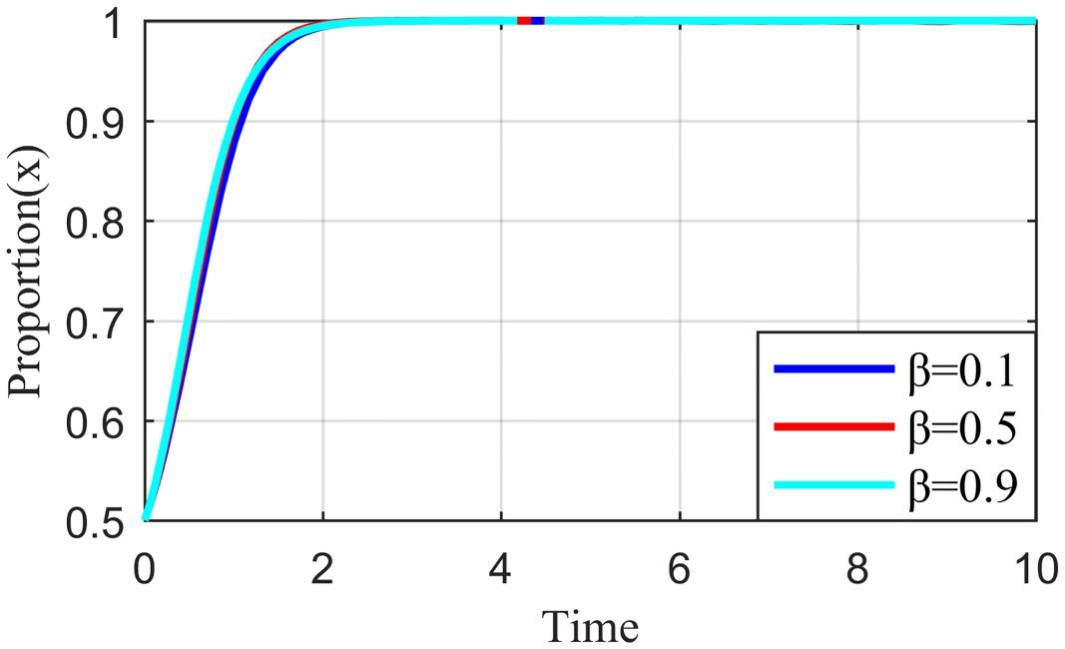
Effect of the revenue allocation coefficient β on live streaming anchor.

**Fig 21:**
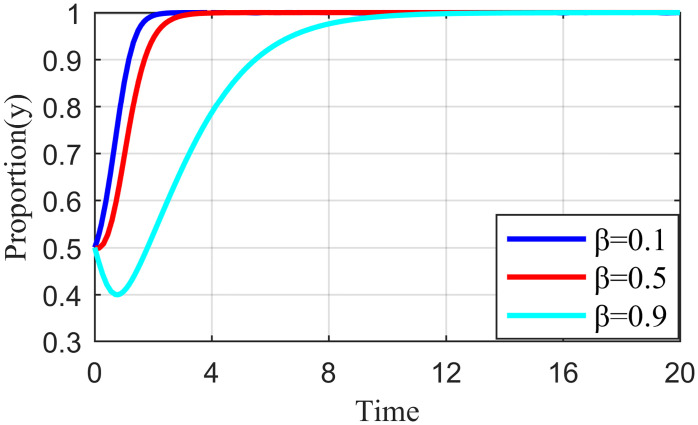
Effect of the revenue allocation coefficient β on supplier.

Fig 20 shows that the strategy choice of the live streaming anchor is not affected by *β*. When *β* = 0.1, the external revenue allocation weight of the supplier is significant and the supplier with the high revenue gained will quickly choose the honesty behavior strategy. As *β* increases, the external revenue allocation weight of the supplier gradually becomes smaller, the gains obtained keep decreasing, and the time required for suppliers to make an honesty behavior strategy becomes longer, such as in Fig 21. When *β* = 0.1, the network externality gain allocation factor of the live streaming platform is small, the network externality gain of the live streaming platform is also small, and the live streaming platform may have a speculative mentality in the behavior strategy selection. In addition, the evolution time required for the live streaming platform to tend to stabilize the strategy at this time is the longest, and when *β* = 0.9, the network externality gain of the live streaming platform is the largest. Additionally, the shortest time required for the live streaming platform to converge to a strict regulatory stabilization strategy.

### 4.4. Discussion

By constructing a tripartite evolutionary game model with the anchor, the supplier, and the live streaming platform, this study initially analyzed the current solutions for value co-creation in the live streaming ecosystem. In addition, the influences of three typical parameters on the live streaming ecosystem, including incentive strength, punishment intensity, and network externality benefit distribution, were investigated in this study. According to the results and analyses presented in Section 4.3, the three parameters influenced on the three participants under different strategy choices. In terms of the intensity of rewards and penalties, the probability of live streaming anchors and suppliers choosing a breach of trust or rent-seeking behavior strategy decreases as the intensity of rewards and penalties increases, which is accordance with previous findings [1, 38]. Most existing research on live streaming only studied two subjects: live streaming anchors and live streaming platforms. In addition, the existing studies typically used simplified initial parameters that were far different from actual conditions, rendering their results limited and lacking practical reference meaning. As this study developed a new model considering the effects of incentive strength, punishment intensity, and network externality benefit distribution on promoting the sustainable development of live streaming industry, the analysis results, and the policy and technical recommendations of this study, will have more practical reference value.

This study considers value co-creation in the live streaming platform ecosystem based on perspectives in previous research. Although value co-creation theory has been used in a variety of areas, such as healthcare, social media, travel, public services and retail [33–37], research on value co-creation in the live streaming platforms ecosystem is limited. Considering sustainable development, the platform will adopt rewards and punishment mechanisms for live streaming anchors and suppliers in live streaming. Under the strict supervision of the live streaming platform, live streaming anchors and suppliers will eventually choose to act in good faith driven by profits, thus, realizing value co-creation. Therefore, this study enriches the main content of live streaming and accelerates the value co-creation process in a live streaming ecosystem.

## 5. Conclusions and policy implications

This study considered people’s limited capacity to be rational and built a tripartite evolutionary game model with the live streaming anchor, the supplier, and the live streaming platform. In addition, this paper analyzed the evolutionary stabilization strategies and investigated how the live streaming platform can control the violations existing in live streaming through a reasonable policy strategy to reduce the problems of false promotion and false traffic, purify the environment of live streaming, maintain the possibility of purifying the live webcast environment, maintain social credit, and protect the legal rights of citizens. The authenticity and validity of the research content were verified using numerical simulation analysis. The following results were obtained:

1. The difference in the initial probability had no effect on the stabilization strategy of the system but rather only affected the time at which the system became stable. When live streaming platforms are strictly regulated, live streaming anchors and suppliers will be attracted to honesty driven by prospective profits, resulting in honesty behaviors being stable in the market. ESS is only influenced by the values of parameters that satisfy the stability conditions. The larger the initial value is, the faster the evolutionary trajectory reaches the ESS (1,1,1).
2. The strength of the reward and punishment can affect the size of the co-creation value. Live streaming platform behavior will appear shocking when the strength of the reward is too large, thus hindering the importance of the co-creation effect of the ecological system of the live streaming platform. As the incentive strength rises from 0.3 to 0.6, the time required for live streaming anchors and suppliers to reach a stable strategy is shortened and the rate increases; however, the strategy of the live streaming platforms adjusts more slowly and takes longer to stabilize. This may be related to the negative impact of incentive strength on live streaming platform revenue. However, the live streaming platform side needs to be brave enough to shoulder the social responsibility in its revenue and cultivate a good live streaming ecology. When the incentive reaches 0.8, the live streaming anchor still chooses the integrity strategy, but the strategy choices of the supplier and the live streaming platform adopt an oscillation phenomenon. The system cannot reach a stable state, and the value probability of co-destruction increases. Moreover, as the penalties increase, live streaming anchors and suppliers will choose integrity strategies more quickly, and the supplier is relatively sensitive to the punishment intensity.
3. An increase in the revenue distribution coefficient has a positive effect on the value co-creation of the ecological system of the live streaming platform. The live streaming anchor is relatively the least sensitive to changes in distribution coefficients.

The study of evolutionary game in terms of value co-creation in the ecosystem of live streaming platforms provides the following suggestions.

1. Establish a scientific reward and punishment mechanism, reasonably allocate the network externality gain coefficient, and promote value co-creation. Although an increase in reward and punishment strengths can improve the evolutionary efficiency of the live streaming anchor and the supplier tending to an honesty strategy and reduce the probability of collusion events, when the reward strength is too high, the live streaming platform cost burden is too heavy. Such conditions prevent the live streaming platform from operating sustainably and benignly, leading to unstable strategic choices for each subject. At the same time, excessively high penalties will require the live streaming platform to pay more human and financial resources, which will also limit the governance efficiency of the live streaming platform. Moreover, increasing the revenue allocation coefficient of the network externality will also promote the stability of the positive strategies of each subject. When the revenue allocation coefficient is too small, the live streaming platform will consider a negative regulatory strategy because of the pursuit of an increase in user scale and traffic. Therefore, the live streaming platform needs to grasp the rewards and punishments further, build a scientific and reasonable reward and punishment system, improve the revenue distribution system based on traffic regulation and evaluation mechanisms, motivate the live streaming anchor to perform an excellent job by actively performing their duties to provide better live streaming content and product services, thus obtaining traffic support and avoiding unfair competition and monopoly phenomena using punishment to maintain the sustainable of the live streaming ecosystem.
2. Building a value co-creation governance system that regulates the behavior of the live streaming anchor. The live streaming anchor is a crucial subject in the ecological system of the live streaming platform. The live streaming platform should appropriately strengthen punishments, optimize punishment measures, and establish a credit scoring system for the live streaming anchor. In the long run, for healthy and sustainable development of the live streaming ecosystem, the live streaming platform should improve the entry threshold for the live streaming anchor and strengthen the pre-broadcast audit mechanism to enhance implementing incentives.
3. Establish a supplier blocklist system and withdrawal mechanism. To establish a supplier qualification verification mechanism, name registration and qualification audits of suppliers must be accurate, industry associations must be relevant, and administrative departments must share or exchange information related to the administrative penalties of suppliers. The supplier information changes should be updated promptly and a blocklist system and withdrawal mechanism of the supplier must be established. In the event of violations of laws and regulations or live streaming platform rules and other circumstances, warning reminders are required for the deadline for rectification and other measures. The accounts of suppliers who commit severe violations of the law or have destructive social impacts should be placed on the blocklist and canceled if necessary.
4. Strengthen the social responsibility of the live streaming platform, clarify the leadership responsibility of ecosystem value co-creation governance, and reduce the risk of value co-destruction. The strategy choice of the live streaming platform side has a crucial influence on the behavior of the anchor and the supplier. Although when the live streaming platform chooses to supervise strictly to a certain extent, which will increase the supervision cost and the risk of decreasing user scale and user traffic, its strategy choice of strict supervision can guide live streaming anchors toward adopting ethical behaviors and give up behaviors that will hinder value co-creation, such as false promotion. This strategy can guide and encourage suppliers to give up substandard and dishonest behaviors. Therefore, the live streaming platform itself should take the initiative to assume social responsibility, play the leading role, strengthen public opinion against false promotion, enhance user education, guide the live streaming anchor and the supplier to use resources rationally and interact efficiently, catalyze the aggregation of all subjects in the live streaming platform to face the problems together, and stimulate bilateral users in the live streaming platform to fulfill their responsibilities. There are three key parameters in this problem, including the strength of the reward and punishment and the network externality benefit distribution. The live streaming anchors, suppliers, and live streaming platforms will make different strategic choices based on parameter changes. Thus, by adjusting these parameters, value co-creation can be achieved in the live streaming ecosystem. These results can also inform government agencies or live streaming platforms to consider these three key parameters when designing policies/business models.

This study has certain limitations: (1) The subjects involved were only the tripartite groups of the live streaming anchors, suppliers, and live streaming platform; however, consumers also influence the live streaming ecosystem during the live streaming process. (2) Although the relevant influencing factors were simulated and analyzed in this study, more factors should be studied, such as the embeddedness of the live streaming anchor and the supplier. Therefore, future studies can solve these problems by constructing a four parties game model including the consumers and analyzing the embeddedness of the live streaming anchor to make the results more consistent with reality and to enhance their objectivity and usability.

## Supporting information

S1 File (DOCX)
