# Supplementary material for: The evolutionary game analysis of the decision-making behavior of live streaming stakeholders under the value co-creation
Source: PLoS One. 2023 Sep 22;18(9):e0291453. doi: 10.1371/journal.pone.0291453 (PMC10516438; doi:10.1371/journal.pone.0291453)
Supplement: S1 File — (DOCX) [file pone.0291453.s001.docx]

**MATLAB 2016b**

**Figure 2:**

**zhubo.m**

function dydt=zhubo(t,y,Ea,Es,Ep,Ca,Cs,Cp,Ia,Is,alpha,beta,lamda,Ra,Rs,theta,Fa,Fs,gamma,Da,Ds) dydt=zeros(3,1);

dydt(1)=-y(1)*(y(1)-1)*(Ia*y(3)-Ca-Ia*alpha*y(3)-Da*gamma*y(3)+Fa*theta*y(3)+Ra*lamda*y(3)+Da*gamma*y(2)*y(3));

dydt(2)=-y(2)*(y(2)-1)*(Is*y(3)-Cs-Ds*gamma*y(3)-Is*beta*y(3)+Fs*theta*y(3)+Rs*lamda*y(3)+Ds*gamma*y(1)*y(3));

dydt(3)=y(3)*(y(3)-1)*(Cp-Fa*theta-Fs*theta-Ia*alpha*y(1)-Is*beta*y(2)+Fa*theta*y(1)+Fs*theta*y(2)+Ra*lamda*y(1)+Rs*lamda*y(2));

end

**jibenfangzhen.m**

clc,clear;

figure(2a);

Ea=2,Es=6,Ep=1,Ca=5,Cs=5,Cp=6,Ia=5,Is=4,alpha=0.5,beta=0.5,Ra=3,Rs=1,lamda=0.3,gamma=0.5,Fa=6,Fs=4,theta=0.5,Da=2,Ds=6;

for i=0.1:0.2:1

for j=0.1:0.2:1

for k=0.1:0.2:1

[t,y]=ode45(@(t,y) zhubo(t,y,Ea,Es,Ep,Ca,Cs,Cp,Ia,Is,alpha,beta,lamda,Ra,Rs,theta,Fa,Fs,gamma,Da,Ds),[0 50],[i j k]);

plot3(y(:,1),y(:,2),y(:,3),'linewidth',1);

set(gca,'XTick',[0:0.2:1],'YTick',[0:0.2:1],'ZTick',[0:0.2:1])

hold on

axis([0 1 0 1 0 1])

view([45 10])

end

end

end

grid on

hold on

xlabel('x','Rotation',0);

ylabel('y','Rotation',0);

zlabel('z','Rotation',360,'position',[0 0 1.05]);

clc,clear;

figure(2b);

Ea=2,Es=6,Ep=1,Ca=6,Cs=5,Cp=4,Ia=5,Is=4,alpha=0.5,beta=0.5,Ra=3,Rs=1,lamda=0.3,gamma=0.5,Fa=6,Fs=4,theta=0.5,Da=2,Ds=6;

for i=0.1:0.2:1

for j=0.1:0.2:1

for k=0.1:0.2:1

[t,y]=ode45(@(t,y) zhubo(t,y,Ea,Es,Ep,Ca,Cs,Cp,Ia,Is,alpha,beta,lamda,Ra,Rs,theta,Fa,Fs,gamma,Da,Ds),[0 50],[i j k]);

plot3(y(:,1),y(:,2),y(:,3),'linewidth',1);

set(gca,'XTick',[0:0.2:1],'YTick',[0:0.2:1],'ZTick',[0:0.2:1])

hold on

axis([0 1 0 1 0 1])

view([45 10])

end

end

end

grid on

hold on

xlabel('x','Rotation',0);

ylabel('y','Rotation',0);

zlabel('z','Rotation',360,'position',[0 0 1.05]);

clc,clear;

figure(2c);

Ea=2,Es=6,Ep=1,Ca=5,Cs=3,Cp=4,Ia=3,Is=8,alpha=0.5,beta=0.5,Ra=3,Rs=1,lamda=0.3,gamma=0.5,Fa=6,Fs=4,theta=0.5,Da=2,Ds=6;

for i=0.1:0.2:1

for j=0.1:0.2:1

for k=0.1:0.2:1

[t,y]=ode45(@(t,y) zhubo(t,y,Ea,Es,Ep,Ca,Cs,Cp,Ia,Is,alpha,beta,lamda,Ra,Rs,theta,Fa,Fs,gamma,Da,Ds),[0 50],[i j k]);

plot3(y(:,1),y(:,2),y(:,3),'linewidth',1);

set(gca,'XTick',[0:0.2:1],'YTick',[0:0.2:1],'ZTick',[0:0.2:1])

hold on

axis([0 1 0 1 0 1])

view([45 10])

end

end

end

grid on

hold on

xlabel('x','Rotation',0);

ylabel('y','Rotation',0);

zlabel('z','Rotation',360,'position',[0 0 1.05]);

clc,clear;

figure(2d);

Ea=2,Es=6,Ep=1,Ca=1,Cs=5,Cp=2,Ia=5,Is=4,alpha=0.5,beta=0.5,Ra=3,Rs=1,lamda=0.3,gamma=0.5,Fa=6,Fs=4,theta=0.5,Da=2,Ds=6;

for i=0.1:0.2:1

for j=0.1:0.2:1

for k=0.1:0.2:1

[t,y]=ode45(@(t,y) zhubo(t,y,Ea,Es,Ep,Ca,Cs,Cp,Ia,Is,alpha,beta,lamda,Ra,Rs,theta,Fa,Fs,gamma,Da,Ds),[0 50],[i j k]);

plot3(y(:,1),y(:,2),y(:,3),'linewidth',1);

set(gca,'XTick',[0:0.2:1],'YTick',[0:0.2:1],'ZTick',[0:0.2:1])

hold on

axis([0 1 0 1 0 1])

view([45 10])

end

end

end

grid on

hold on

xlabel('x','Rotation',0);

ylabel('y','Rotation',0);

zlabel('z','Rotation',360,'position',[0 0 1.05]);

clc,clear;

figure(2e);

Ea=2,Es=6,Ep=1,Ca=1,Cs=1,Cp=2,Ia=5,Is=4,alpha=0.5,beta=0.5,Ra=3,Rs=1,lamda=0.3,gamma=0.5,Fa=4,Fs=2,theta=0.5,Da=2,Ds=6;

for i=0.1:0.2:1

for j=0.1:0.2:1

for k=0.1:0.2:1

[t,y]=ode45(@(t,y) zhubo(t,y,Ea,Es,Ep,Ca,Cs,Cp,Ia,Is,alpha,beta,lamda,Ra,Rs,theta,Fa,Fs,gamma,Da,Ds),[0 50],[i j k]);

plot3(y(:,1),y(:,2),y(:,3),'linewidth',1);

set(gca,'XTick',[0:0.2:1],'YTick',[0:0.2:1],'ZTick',[0:0.2:1])

hold on

axis([0 1 0 1 0 1])

view([45 10])

end

end

end

grid on

hold on

xlabel('x','Rotation',0);

ylabel('y','Rotation',0);

zlabel('z','Rotation',360,'position',[0 0 1.05]);

**Figure 3:**

**differential.m**

function dxdt=differential(t,x)

Ea=2;Es=6;Ep=1;Ca=1;Cs=1;Cp=2;Ia=5;Is=4;alpha=0.5;beta=0.5;Ra=3;Rs=1;lamda=0.3;gamma=0.5;Fa=4;Fs=2;theta=0.5;Da=2;Ds=6;

dxdt=[-x(1)*(x(1)-1)*(Ia*x(3)-Ca-Ia*alpha*x(3)-Da*gamma*x(3)+Fa*theta*x(3)+Ra*lamda*x(3)+Da*gamma*x(2)*x(3));

-x(2)*(x(2)-1)*(Is*x(3)-Cs-Ds*gamma*x(3)-Is*beta*x(3)+Fs*theta*x(3)+Rs*lamda*x(3)+Ds*gamma*x(1)*x(3));

x(3)*(x(3)-1)*(Cp-Fa*theta-Fs*theta-Ia*alpha*x(1)-Is*beta*x(2)+Fa*theta*x(1)+Fs*theta*x(2)+Ra*lamda*x(1)+Rs*lamda*x(2))];

end

**untitled.m**

clc,clear;

for i=0.2

for j=0.2

for k=0.2

[T,Y]=ode45('differential',[0 10],[i j k]);

figure(3a)

grid on

plot(T,Y(:,1),'b','linewidth',1.5);

hold on

plot(T,Y(:,2),'r','linewidth',1.5);

hold on

plot(T,Y(:,3),'c','linewidth',1.5);

hold on

xlim([0,10])

set(0,'defaultfigurecolor','w')

grid on

hold on

ylim([0,1])

xlabel('Time');

ylabel('Proportion');

set(get(gca,'xlabel'),'FontName', 'Times New Roman','FontSize',12);

set(get(gca,'ylabel'),'FontName', 'Times New Roman','FontSize',12);

set(gca,'ytick',[0,0.2,0.4,0.6,0.8,1]);

end

end

end

legend('Anchor','Supplier','Platform');

clc,clear;

for i=0.5

for j=0.5

for k=0.5

[T,Y]=ode45('differential',[0 10],[i j k]);

figure(3b)

grid on

plot(T,Y(:,1),'b','linewidth',1.5);

hold on

plot(T,Y(:,2),'r','linewidth',1.5);

hold on

plot(T,Y(:,3),'c','linewidth',1.5);

hold on

set(0,'defaultfigurecolor','w')

grid on

hold on

set(gca,'xtick');

ylim([0.5,1])

xlabel('Time');

ylabel('Proportion');

set(get(gca,'xlabel'),'FontName', 'Times New Roman','FontSize',12);

set(get(gca,'ylabel'),'FontName', 'Times New Roman','FontSize',12);

set(gca,'ytick',[0.5,0.6,0.7,0.8,0.9,1]);

end

end

end

legend('Anchor','Supplier','Platform');

clc,clear;

for i=0.8

for j=0.8

for k=0.8

[T,Y]=ode45('differential',[0 10],[i j k]);

figure(3c)

grid on

plot(T,Y(:,1),'b','linewidth',1.5);

hold on

plot(T,Y(:,2),'r','linewidth',1.5);

hold on

plot(T,Y(:,3),'c','linewidth',1.5);

hold on

xlim([0,10])

set(0,'defaultfigurecolor','w')

grid on

hold on

ylim([0.8,1])

xlabel('Time');

ylabel('Proportion');

set(get(gca,'xlabel'),'FontName', 'Times New Roman','FontSize',12);

set(get(gca,'ylabel'),'FontName', 'Times New Roman','FontSize',12);

set(gca,'ytick',[0.8,0.9,1]);

end

end

end

legend('Anchor','Supplier','Platform');

**Figure 4:**

**zhubo.m**

function dydt=zhubo(t,y,Ea,Es,Ep,Ca,Cs,Cp,Ia,Is,alpha,beta,lamda,Ra,Rs,theta,Fa,Fs,gamma,Da,Ds)

dydt=zeros(3,1);

dydt(1)=-y(1)*(y(1)-1)*(Ia*y(3)-Ca-Ia*alpha*y(3)-Da*gamma*y(3)+Fa*theta*y(3)+Ra*lamda*y(3)+Da*gamma*y(2)*y(3));

dydt(2)=-y(2)*(y(2)-1)*(Is*y(3)-Cs-Ds*gamma*y(3)-Is*beta*y(3)+Fs*theta*y(3)+Rs*lamda*y(3)+Ds*gamma*y(1)*y(3));

dydt(3)=y(3)*(y(3)-1)*(Cp-Fa*theta-Fs*theta-Ia*alpha*y(1)-Is*beta*y(2)+Fa*theta*y(1)+Fs*theta*y(2)+Ra*lamda*y(1)+Rs*lamda*y(2));

end

**lamda.m**

Ea=2,Es=6,Ep=1,Ca=1,Cs=1,Cp=1,Ia=5,Is=4,alpha=0.6,beta=0.5,lamda=0.2,Ra=3,Rs=1,theta=0.5,Fa=4,Fs=2,gamma=0.5,Da=2,Ds=6;

clc,clear;

figure(4a);

Ea=2,Es=6,Ep=1,Ca=1,Cs=1,Cp=1,Ia=5,Is=4,alpha=0.5,beta=0.5,Ra=3,Rs=1,lamda=0.3,gamma=0.5,Fa=4,Fs=2,theta=0.5,Da=2,Ds=6;

[t,y]=ode45(@(t,y) zhubo(t,y,Ea,Es,Ep,Ca,Cs,Cp,Ia,Is,alpha,beta,lamda,Ra,Rs,theta,Fa,Fs,gamma,Da,Ds),[0 10],[0.5 0.5 0.5]);

points=1:1:length(t);

plot(t,y(:,1),'b','linewidth',1.5);

hold on;

Ea=2,Es=6,Ep=1,Ca=1,Cs=1,Cp=1,Ia=5,Is=4,alpha=0.5,beta=0.5,Ra=3,Rs=1,lamda=0.6,gamma=0.5,Fa=4,Fs=2,theta=0.5,Da=2,Ds=6;

[t,y]=ode45(@(t,y) zhubo(t,y,Ea,Es,Ep,Ca,Cs,Cp,Ia,Is,alpha,beta,lamda,Ra,Rs,theta,Fa,Fs,gamma,Da,Ds),[0 10],[0.5 0.5 0.5]);

points=1:1:length(t);

plot(t,y(:,1),'r','linewidth',1.5);

hold on;

Ea=2,Es=6,Ep=1,Ca=1,Cs=1,Cp=1,Ia=5,Is=4,alpha=0.6,beta=0.5,lamda=0.2,Ra=3,Rs=1,theta=0.5,Fa=4,Fs=2,gamma=0.5,Da=2,Ds=6;

[t,y]=ode45(@(t,y) zhubo(t,y,Ea,Es,Ep,Ca,Cs,Cp,Ia,Is,alpha,beta,lamda,Ra,Rs,theta,Fa,Fs,gamma,Da,Ds),[0 10],[0.5 0.5 0.5]);

points=1:1:length(t);

plot(t,y(:,1),'c','linewidth',1.5);

hold on;

set(0,'defaultfigurecolor','w')

grid on

hold on

xlabel('Time');

ylabel('Proportion(x)');

set(get(gca,'xlabel'),'FontName', 'Times New Roman','FontSize',12);

set(get(gca,'ylabel'),'FontName', 'Times New Roman','FontSize',12);

set(gca,'XTick',[0:2:10],'YTick',[0.5:0.1:1])

axis([0 10 0.5 1]);

legend('λ=0.3','λ=0.6','λ=0.9');

figure(4b);

Ea=2,Es=6,Ep=1,Ca=1,Cs=1,Cp=1,Ia=5,Is=4,alpha=0.5,beta=0.5,Ra=3,Rs=1,lamda=0.3,gamma=0.5,Fa=4,Fs=2,theta=0.5,Da=2,Ds=6;

[t,y]=ode45(@(t,y) zhubo(t,y,Ea,Es,Ep,Ca,Cs,Cp,Ia,Is,alpha,beta,lamda,Ra,Rs,theta,Fa,Fs,gamma,Da,Ds),[0 10],[0.5 0.5 0.5]);

points=1:1:length(t);

plot(t,y(:,2),'b','linewidth',1.5);

hold on;

Ea=2,Es=6,Ep=1,Ca=1,Cs=1,Cp=1,Ia=5,Is=4,alpha=0.5,beta=0.5,Ra=3,Rs=1,lamda=0.6,gamma=0.5,Fa=4,Fs=2,theta=0.5,Da=2,Ds=6;

[t,y]=ode45(@(t,y) zhubo(t,y,Ea,Es,Ep,Ca,Cs,Cp,Ia,Is,alpha,beta,lamda,Ra,Rs,theta,Fa,Fs,gamma,Da,Ds),[0 10],[0.5 0.5 0.5]);

points=1:1:length(t);

plot(t,y(:,2),'r','linewidth',1.5);

hold on;

Ea=2,Es=6,Ep=1,Ca=1,Cs=1,Cp=1,Ia=5,Is=4,alpha=0.5,beta=0.5,Ra=3,Rs=1,lamda=0.9,gamma=0.5,Fa=4,Fs=2,theta=0.5,Da=2,Ds=6;

[t,y]=ode45(@(t,y) zhubo(t,y,Ea,Es,Ep,Ca,Cs,Cp,Ia,Is,alpha,beta,lamda,Ra,Rs,theta,Fa,Fs,gamma,Da,Ds),[0 10],[0.5 0.5 0.5]);

points=1:1:length(t);

plot(t,y(:,2),'c','linewidth',1.5);

hold on;

set(0,'defaultfigurecolor','w')

grid on

hold on

xlabel('Time');

ylabel('Proportion(y)');

set(get(gca,'xlabel'),'FontName', 'Times New Roman','FontSize',12);

set(get(gca,'ylabel'),'FontName', 'Times New Roman','FontSize',12);

set(gca,'XTick',[0:2:10],'YTick',[0.5:0.1:1])

axis([0 10 0.5 1]);

legend('λ=0.3','λ=0.6','λ=0.9');

figure(4c);

Ea=2,Es=6,Ep=1,Ca=1,Cs=1,Cp=1,Ia=5,Is=4,alpha=0.5,beta=0.5,Ra=3,Rs=1,lamda=0.3,gamma=0.5,Fa=4,Fs=2,theta=0.5,Da=2,Ds=6;

[t,y]=ode45(@(t,y) zhubo(t,y,Ea,Es,Ep,Ca,Cs,Cp,Ia,Is,alpha,beta,lamda,Ra,Rs,theta,Fa,Fs,gamma,Da,Ds),[0 10],[0.5 0.5 0.5]);

points=1:1:length(t);

plot(t,y(:,3),'b','linewidth',1.5);

hold on;

Ea=2,Es=6,Ep=1,Ca=1,Cs=1,Cp=1,Ia=5,Is=4,alpha=0.5,beta=0.5,Ra=3,Rs=1,lamda=0.6,gamma=0.5,Fa=4,Fs=2,theta=0.5,Da=2,Ds=6;

[t,y]=ode45(@(t,y) zhubo(t,y,Ea,Es,Ep,Ca,Cs,Cp,Ia,Is,alpha,beta,lamda,Ra,Rs,theta,Fa,Fs,gamma,Da,Ds),[0 10],[0.5 0.5 0.5]);

points=1:1:length(t);

plot(t,y(:,3),'r','linewidth',1.5);

hold on;

Ea=2,Es=6,Ep=1,Ca=1,Cs=1,Cp=1,Ia=5,Is=4,alpha=0.5,beta=0.5,Ra=3,Rs=1,lamda=0.9,gamma=0.5,Fa=4,Fs=2,theta=0.5,Da=2,Ds=6;

[t,y]=ode45(@(t,y) zhubo(t,y,Ea,Es,Ep,Ca,Cs,Cp,Ia,Is,alpha,beta,lamda,Ra,Rs,theta,Fa,Fs,gamma,Da,Ds),[0 10],[0.5 0.5 0.5]);

points=1:1:length(t);

plot(t,y(:,3),'c','linewidth',1.5);

hold on;

set(0,'defaultfigurecolor','w')

grid on

hold on

set(gca,'XTick',[0:2:10],'YTick',[0.2:0.1:1])

xlabel('Time');

ylabel('Proportion(z)');

set(get(gca,'xlabel'),'FontName', 'Times New Roman','FontSize',12);

set(get(gca,'ylabel'),'FontName', 'Times New Roman','FontSize',12);

axis([0 10 0.2 1]);

legend('λ=0.3','λ=0.6','λ=0.9');

**Figure 5:**

**zhubo.m**

function dydt=zhubo(t,y,Ea,Es,Ep,Ca,Cs,Cp,Ia,Is,alpha,beta,lamda,Ra,Rs,theta,Fa,Fs,gamma,Da,Ds)

dydt=zeros(3,1);

dydt(1)=-y(1)*(y(1)-1)*(Ia*y(3)-Ca-Ia*alpha*y(3)-Da*gamma*y(3)+Fa*theta*y(3)+Ra*lamda*y(3)+Da*gamma*y(2)*y(3));

dydt(2)=-y(2)*(y(2)-1)*(Is*y(3)-Cs-Ds*gamma*y(3)-Is*beta*y(3)+Fs*theta*y(3)+Rs*lamda*y(3)+Ds*gamma*y(1)*y(3));

dydt(3)=y(3)*(y(3)-1)*(Cp-Fa*theta-Fs*theta-Ia*alpha*y(1)-Is*beta*y(2)+Fa*theta*y(1)+Fs*theta*y(2)+Ra*lamda*y(1)+Rs*lamda*y(2));

end

**theta.m**

Ea=2,Es=6,Ep=1,Ca=1,Cs=1,Cp=1,Ia=5,Is=4,alpha=0.6,beta=0.5,lamda=0.2,Ra=3,Rs=1,theta=0.5,Fa=4,Fs=2,gamma=0.5,Da=2,Ds=6;

clc,clear;

figure(5a);

Ea=2,Es=6,Ep=1,Ca=1,Cs=1,Cp=1,Ia=5,Is=4,alpha=0.5,beta=0.5,Ra=3,Rs=1,lamda=0.2,gamma=0.5,Fa=4,Fs=2,theta=0.1,Da=2,Ds=6;

[t,y]=ode45(@(t,y) zhubo(t,y,Ea,Es,Ep,Ca,Cs,Cp,Ia,Is,alpha,beta,lamda,Ra,Rs,theta,Fa,Fs,gamma,Da,Ds),[0 10],[0.5 0.5 0.5]);

points=1:1:length(t);

plot(t,y(:,1),'b','linewidth',1.5);

hold on;

Ea=2,Es=6,Ep=1,Ca=1,Cs=1,Cp=1,Ia=5,Is=4,alpha=0.5,beta=0.5,Ra=3,Rs=1,lamda=0.2,gamma=0.5,Fa=4,Fs=2,theta=0.5,Da=2,Ds=6;

[t,y]=ode45(@(t,y) zhubo(t,y,Ea,Es,Ep,Ca,Cs,Cp,Ia,Is,alpha,beta,lamda,Ra,Rs,theta,Fa,Fs,gamma,Da,Ds),[0 10],[0.5 0.5 0.5]);

points=1:1:length(t);

plot(t,y(:,1),'r','linewidth',1.5);

hold on;

Ea=2,Es=6,Ep=1,Ca=1,Cs=1,Cp=1,Ia=5,Is=4,alpha=0.5,beta=0.5,Ra=3,Rs=1,lamda=0.2,gamma=0.5,Fa=4,Fs=2,theta=0.9,Da=2,Ds=6;

[t,y]=ode45(@(t,y) zhubo(t,y,Ea,Es,Ep,Ca,Cs,Cp,Ia,Is,alpha,beta,lamda,Ra,Rs,theta,Fa,Fs,gamma,Da,Ds),[0 10],[0.5 0.5 0.5]);

points=1:1:length(t);

plot(t,y(:,1),'c','linewidth',1.5);

hold on;

set(0,'defaultfigurecolor','w')

grid on

hold on

xlabel('Time');

ylabel('Proportion(x)');

set(get(gca,'xlabel'),'FontName', 'Times New Roman','FontSize',12);

set(get(gca,'ylabel'),'FontName', 'Times New Roman','FontSize',12);

set(gca,'XTick',[0:2:10],'YTick',[0.5:0.1:1])

axis([0 10 0.5 1]);

legend('θ=0.1', 'θ=0.5 ', 'θ=0.9');

figure(5b);

Ea=2,Es=6,Ep=1,Ca=1,Cs=1,Cp=1,Ia=5,Is=4,alpha=0.5,beta=0.5,Ra=3,Rs=1,lamda=0.2,gamma=0.5,Fa=4,Fs=2,theta=0.1,Da=2,Ds=6;

[t,y]=ode45(@(t,y) zhubo(t,y,Ea,Es,Ep,Ca,Cs,Cp,Ia,Is,alpha,beta,lamda,Ra,Rs,theta,Fa,Fs,gamma,Da,Ds),[0 10],[0.5 0.5 0.5]);

points=1:1:length(t);

plot(t,y(:,2),'b','linewidth',1.5);

hold on;

Ea=2,Es=6,Ep=1,Ca=1,Cs=1,Cp=1,Ia=5,Is=4,alpha=0.5,beta=0.5,Ra=3,Rs=1,lamda=0.2,gamma=0.5,Fa=4,Fs=2,theta=0.5,Da=2,Ds=6;

[t,y]=ode45(@(t,y) zhubo(t,y,Ea,Es,Ep,Ca,Cs,Cp,Ia,Is,alpha,beta,lamda,Ra,Rs,theta,Fa,Fs,gamma,Da,Ds),[0 10],[0.5 0.5 0.5]);

points=1:1:length(t);

plot(t,y(:,2),'r','linewidth',1.5);

hold on;

Ea=2,Es=6,Ep=1,Ca=1,Cs=1,Cp=1,Ia=5,Is=4,alpha=0.5,beta=0.5,Ra=3,Rs=1,lamda=0.2,gamma=0.5,Fa=4,Fs=2,theta=0.9,Da=2,Ds=6;

[t,y]=ode45(@(t,y) zhubo(t,y,Ea,Es,Ep,Ca,Cs,Cp,Ia,Is,alpha,beta,lamda,Ra,Rs,theta,Fa,Fs,gamma,Da,Ds),[0 10],[0.5 0.5 0.5]);

points=1:1:length(t);

plot(t,y(:,2),'c','linewidth',1.5);

hold on;

set(0,'defaultfigurecolor','w')

grid on

hold on

xlabel('Time');

ylabel('Proportion(y)');

set(get(gca,'xlabel'),'FontName', 'Times New Roman','FontSize',12);

set(get(gca,'ylabel'),'FontName', 'Times New Roman','FontSize',12);

set(gca,'XTick',[0:2:10],'YTick',[0.4:0.1:1])

axis([0 10 0.4 1]);

legend('θ=0.1', 'θ=0.5 ', 'θ=0.9');

figure(5c);

Ea=2,Es=6,Ep=1,Ca=1,Cs=1,Cp=1,Ia=5,Is=4,alpha=0.5,beta=0.5,Ra=3,Rs=1,lamda=0.2,gamma=0.5,Fa=4,Fs=2,theta=0.1,Da=2,Ds=6;

[t,y]=ode45(@(t,y) zhubo(t,y,Ea,Es,Ep,Ca,Cs,Cp,Ia,Is,alpha,beta,lamda,Ra,Rs,theta,Fa,Fs,gamma,Da,Ds),[0 10],[0.5 0.5 0.5]);

points=1:1:length(t);

plot(t,y(:,3),'b','linewidth',1.5);

hold on;

Ea=2,Es=6,Ep=1,Ca=1,Cs=1,Cp=1,Ia=5,Is=4,alpha=0.5,beta=0.5,Ra=3,Rs=1,lamda=0.2,gamma=0.5,Fa=4,Fs=2,theta=0.5,Da=2,Ds=6;

[t,y]=ode45(@(t,y) zhubo(t,y,Ea,Es,Ep,Ca,Cs,Cp,Ia,Is,alpha,beta,lamda,Ra,Rs,theta,Fa,Fs,gamma,Da,Ds),[0 10],[0.5 0.5 0.5]);

points=1:1:length(t);

plot(t,y(:,3),'r','linewidth',1.5);

hold on;

Ea=2,Es=6,Ep=1,Ca=1,Cs=1,Cp=1,Ia=5,Is=4,alpha=0.5,beta=0.5,Ra=3,Rs=1,lamda=0.2,gamma=0.5,Fa=4,Fs=2,theta=0.9,Da=2,Ds=6;

[t,y]=ode45(@(t,y) zhubo(t,y,Ea,Es,Ep,Ca,Cs,Cp,Ia,Is,alpha,beta,lamda,Ra,Rs,theta,Fa,Fs,gamma,Da,Ds),[0 10],[0.5 0.5 0.5]);

points=1:1:length(t);

plot(t,y(:,3),'c','linewidth',1.5);

hold on;

set(0,'defaultfigurecolor','w')

grid on

hold on

set(gca,'XTick',[0:2:10],'YTick',[0.5:0.1:1])

xlabel('Time');

ylabel('Proportion(z)');

set(get(gca,'xlabel'),'FontName', 'Times New Roman','FontSize',12);

set(get(gca,'ylabel'),'FontName', 'Times New Roman','FontSize',12);

axis([0 10 0.5 1]);

legend('θ=0.1', 'θ=0.5 ', 'θ=0.9');

**Figure 6:**

**zhubo.m**

function dydt=zhubo(t,y,Ea,Es,Ep,Ca,Cs,Cp,Ia,Is,alpha,beta,lamda,Ra,Rs,theta,Fa,Fs,gamma,Da,Ds)

dydt=zeros(3,1);

dydt(1)=-y(1)*(y(1)-1)*(Ia*y(3)-Ca-Ia*alpha*y(3)-Da*gamma*y(3)+Fa*theta*y(3)+Ra*lamda*y(3)+Da*gamma*y(2)*y(3));

dydt(2)=-y(2)*(y(2)-1)*(Is*y(3)-Cs-Ds*gamma*y(3)-Is*beta*y(3)+Fs*theta*y(3)+Rs*lamda*y(3)+Ds*gamma*y(1)*y(3));

dydt(3)=y(3)*(y(3)-1)*(Cp-Fa*theta-Fs*theta-Ia*alpha*y(1)-Is*beta*y(2)+Fa*theta*y(1)+Fs*theta*y(2)+Ra*lamda*y(1)+Rs*lamda*y(2));

end

**alpha.m**

Ea=2,Es=6,Ep=1,Ca=1,Cs=1,Cp=1,Ia=5,Is=4,alpha=0.6,beta=0.5,lamda=0.2,Ra=3,Rs=1,theta=0.5,Fa=4,Fs=2,gamma=0.5,Da=2,Ds=6;

clc,clear;

figure(6a);

Ea=2,Es=6,Ep=1,Ca=1,Cs=1,Cp=1,Ia=5,Is=4,alpha=0.1,beta=0.5,Ra=3,Rs=1,lamda=0.2,gamma=0.5,Fa=4,Fs=2,theta=0.5,Da=2,Ds=6;

[t,y]=ode45(@(t,y) zhubo(t,y,Ea,Es,Ep,Ca,Cs,Cp,Ia,Is,alpha,beta,lamda,Ra,Rs,theta,Fa,Fs,gamma,Da,Ds),[0 10],[0.5 0.5 0.5]);

points=1:1:length(t);

plot(t,y(:,1),'b','linewidth',1.5);

hold on;

Ea=2,Es=6,Ep=1,Ca=1,Cs=1,Cp=1,Ia=5,Is=4,alpha=0.5,beta=0.5,Ra=3,Rs=1,lamda=0.2,gamma=0.5,Fa=4,Fs=2,theta=0.5,Da=2,Ds=6;

[t,y]=ode45(@(t,y) zhubo(t,y,Ea,Es,Ep,Ca,Cs,Cp,Ia,Is,alpha,beta,lamda,Ra,Rs,theta,Fa,Fs,gamma,Da,Ds),[0 10],[0.5 0.5 0.5]);

points=1:1:length(t);

plot(t,y(:,1),'r','linewidth',1.5);

hold on;

Ea=2,Es=6,Ep=1,Ca=1,Cs=1,Cp=1,Ia=5,Is=4,alpha=0.9,beta=0.5,Ra=3,Rs=1,lamda=0.2,gamma=0.5,Fa=4,Fs=2,theta=0.5,Da=2,Ds=6;

[t,y]=ode45(@(t,y) zhubo(t,y,Ea,Es,Ep,Ca,Cs,Cp,Ia,Is,alpha,beta,lamda,Ra,Rs,theta,Fa,Fs,gamma,Da,Ds),[0 10],[0.5 0.5 0.5]);

points=1:1:length(t);

plot(t,y(:,1),'c','linewidth',1.5);

hold on;

set(0,'defaultfigurecolor','w')

grid on

hold on

xlabel('Time');

ylabel('Proportion(x)');

set(get(gca,'xlabel'),'FontName', 'Times New Roman','FontSize',12);

set(get(gca,'ylabel'),'FontName', 'Times New Roman','FontSize',12);

set(gca,'XTick',[0:2:10],'YTick',[0.5:0.1:1])

axis([0 10 0.5 1]);

legend('α=0.1', 'α=0.5 ', 'α=0.9');

figure(6b);

Ea=2,Es=6,Ep=1,Ca=1,Cs=1,Cp=1,Ia=5,Is=4,alpha=0.1,beta=0.5,Ra=3,Rs=1,lamda=0.2,gamma=0.5,Fa=4,Fs=2,theta=0.5,Da=2,Ds=6;

[t,y]=ode45(@(t,y) zhubo(t,y,Ea,Es,Ep,Ca,Cs,Cp,Ia,Is,alpha,beta,lamda,Ra,Rs,theta,Fa,Fs,gamma,Da,Ds),[0 10],[0.5 0.5 0.5]);

points=1:1:length(t);

plot(t,y(:,2),'b','linewidth',1.5);

hold on;

Ea=2,Es=6,Ep=1,Ca=1,Cs=1,Cp=1,Ia=5,Is=4,alpha=0.5,beta=0.5,Ra=3,Rs=1,lamda=0.2,gamma=0.5,Fa=4,Fs=2,theta=0.5,Da=2,Ds=6;

[t,y]=ode45(@(t,y) zhubo(t,y,Ea,Es,Ep,Ca,Cs,Cp,Ia,Is,alpha,beta,lamda,Ra,Rs,theta,Fa,Fs,gamma,Da,Ds),[0 10],[0.5 0.5 0.5]);

points=1:1:length(t);

plot(t,y(:,2),'r','linewidth',1.5);

hold on;

Ea=2,Es=6,Ep=1,Ca=1,Cs=1,Cp=1,Ia=5,Is=4,alpha=0.9,beta=0.5,Ra=3,Rs=1,lamda=0.2,gamma=0.5,Fa=4,Fs=2,theta=0.5,Da=2,Ds=6;

[t,y]=ode45(@(t,y) zhubo(t,y,Ea,Es,Ep,Ca,Cs,Cp,Ia,Is,alpha,beta,lamda,Ra,Rs,theta,Fa,Fs,gamma,Da,Ds),[0 10],[0.5 0.5 0.5]);

points=1:1:length(t);

plot(t,y(:,2),'c','linewidth',1.5);

hold on;

set(0,'defaultfigurecolor','w')

grid on

hold on

xlabel('Time');

ylabel('Proportion(y)');

set(get(gca,'xlabel'),'FontName', 'Times New Roman','FontSize',12);

set(get(gca,'ylabel'),'FontName', 'Times New Roman','FontSize',12);

set(gca,'XTick',[0:2:10],'YTick',[0.5:0.1:1])

axis([0 10 0.5 1]);

legend('α=0.1', 'α=0.5 ', 'α=0.9');

figure(6c);

Ea=2,Es=6,Ep=1,Ca=1,Cs=1,Cp=1,Ia=5,Is=4,alpha=0.1,beta=0.5,Ra=3,Rs=1,lamda=0.2,gamma=0.5,Fa=4,Fs=2,theta=0.5,Da=2,Ds=6;

[t,y]=ode45(@(t,y) zhubo(t,y,Ea,Es,Ep,Ca,Cs,Cp,Ia,Is,alpha,beta,lamda,Ra,Rs,theta,Fa,Fs,gamma,Da,Ds),[0 10],[0.5 0.5 0.5]);

points=1:1:length(t);

plot(t,y(:,3),'b','linewidth',1.5);

hold on;

Ea=2,Es=6,Ep=1,Ca=1,Cs=1,Cp=1,Ia=5,Is=4,alpha=0.5,beta=0.5,Ra=3,Rs=1,lamda=0.2,gamma=0.5,Fa=4,Fs=2,theta=0.5,Da=2,Ds=6;

[t,y]=ode45(@(t,y) zhubo(t,y,Ea,Es,Ep,Ca,Cs,Cp,Ia,Is,alpha,beta,lamda,Ra,Rs,theta,Fa,Fs,gamma,Da,Ds),[0 10],[0.5 0.5 0.5]);

points=1:1:length(t);

plot(t,y(:,3),'r','linewidth',1.5);

hold on;

Ea=2,Es=6,Ep=1,Ca=1,Cs=1,Cp=1,Ia=5,Is=4,alpha=0.9,beta=0.5,Ra=3,Rs=1,lamda=0.2,gamma=0.5,Fa=4,Fs=2,theta=0.5,Da=2,Ds=6;

[t,y]=ode45(@(t,y) zhubo(t,y,Ea,Es,Ep,Ca,Cs,Cp,Ia,Is,alpha,beta,lamda,Ra,Rs,theta,Fa,Fs,gamma,Da,Ds),[0 10],[0.5 0.5 0.5]);

points=1:1:length(t);

plot(t,y(:,3),'c','linewidth',1.5);

hold on;

set(0,'defaultfigurecolor','w')

grid on

hold on

xlabel('Time');

ylabel('Proportion(z)');

set(get(gca,'xlabel'),'FontName', 'Times New Roman','FontSize',12);

set(get(gca,'ylabel'),'FontName', 'Times New Roman','FontSize',12);

set(gca,'XTick',[0:2:10],'YTick',[0.5:0.1:1])

axis([0 10 0.5 1]);

legend('α=0.1', 'α=0.5 ', 'α=0.9');

**Figure 7:**

**zhubo.m**

function dydt=zhubo(t,y,Ea,Es,Ep,Ca,Cs,Cp,Ia,Is,alpha,beta,lamda,Ra,Rs,theta,Fa,Fs,gamma,Da,Ds)

dydt=zeros(3,1);

dydt(1)=-y(1)*(y(1)-1)*(Ia*y(3)-Ca-Ia*alpha*y(3)-Da*gamma*y(3)+Fa*theta*y(3)+Ra*lamda*y(3)+Da*gamma*y(2)*y(3));

dydt(2)=-y(2)*(y(2)-1)*(Is*y(3)-Cs-Ds*gamma*y(3)-Is*beta*y(3)+Fs*theta*y(3)+Rs*lamda*y(3)+Ds*gamma*y(1)*y(3));

dydt(3)=y(3)*(y(3)-1)*(Cp-Fa*theta-Fs*theta-Ia*alpha*y(1)-Is*beta*y(2)+Fa*theta*y(1)+Fs*theta*y(2)+Ra*lamda*y(1)+Rs*lamda*y(2));

end

**beta.m**

Ea=2,Es=6,Ep=1,Ca=1,Cs=1,Cp=1,Ia=5,Is=4,alpha=0.6,beta=0.5,lamda=0.2,Ra=3,Rs=1,theta=0.5,Fa=4,Fs=2,gamma=0.5,Da=2,Ds=6;

clc,clear;

figure(7a);

Ea=2,Es=6,Ep=1,Ca=1,Cs=1,Cp=1,Ia=5,Is=4,alpha=0.6,beta=0.1,Ra=3,Rs=1,lamda=0.2,gamma=0.5,Fa=4,Fs=2,theta=0.5,Da=2,Ds=6;

[t,y]=ode45(@(t,y) zhubo(t,y,Ea,Es,Ep,Ca,Cs,Cp,Ia,Is,alpha,beta,lamda,Ra,Rs,theta,Fa,Fs,gamma,Da,Ds),[0 10],[0.5 0.5 0.5]);

points=1:1:length(t);

plot(t,y(:,1),'b','linewidth',1.5);

hold on;

Ea=2,Es=6,Ep=1,Ca=1,Cs=1,Cp=1,Ia=5,Is=4,alpha=0.6,beta=0.5,Ra=3,Rs=1,lamda=0.2,gamma=0.5,Fa=4,Fs=2,theta=0.5,Da=2,Ds=6;

[t,y]=ode45(@(t,y) zhubo(t,y,Ea,Es,Ep,Ca,Cs,Cp,Ia,Is,alpha,beta,lamda,Ra,Rs,theta,Fa,Fs,gamma,Da,Ds),[0 10],[0.5 0.5 0.5]);

points=1:1:length(t);

plot(t,y(:,1),'r','linewidth',1.5);

hold on;

Ea=2,Es=6,Ep=1,Ca=1,Cs=1,Cp=1,Ia=5,Is=4,alpha=0.6,beta=0.9,Ra=3,Rs=1,lamda=0.2,gamma=0.5,Fa=4,Fs=2,theta=0.5,Da=2,Ds=6;

[t,y]=ode45(@(t,y) zhubo(t,y,Ea,Es,Ep,Ca,Cs,Cp,Ia,Is,alpha,beta,lamda,Ra,Rs,theta,Fa,Fs,gamma,Da,Ds),[0 10],[0.5 0.5 0.5]);

points=1:1:length(t);

plot(t,y(:,1),'c','linewidth',1.5);

hold on;

set(0,'defaultfigurecolor','w')

grid on

hold on

xlabel('Time');

ylabel('Proportion(x)');

set(get(gca,'xlabel'),'FontName', 'Times New Roman','FontSize',12);

set(get(gca,'ylabel'),'FontName', 'Times New Roman','FontSize',12);

set(gca,'XTick',[0:2:10],'YTick',[0.5:0.1:1])

axis([0 10 0.5 1]);

legend('β=0.1', 'β=0.5 ', 'β=0.9');

figure(7b);

Ea=2,Es=6,Ep=1,Ca=1,Cs=1,Cp=1,Ia=5,Is=4,alpha=0.6,beta=0.1,Ra=3,Rs=1,lamda=0.2,gamma=0.5,Fa=4,Fs=2,theta=0.5,Da=2,Ds=6;

[t,y]=ode45(@(t,y) zhubo(t,y,Ea,Es,Ep,Ca,Cs,Cp,Ia,Is,alpha,beta,lamda,Ra,Rs,theta,Fa,Fs,gamma,Da,Ds),[0 20],[0.5 0.5 0.5]);

points=1:1:length(t);

plot(t,y(:,2),'b','linewidth',1.5);

hold on;

Ea=2,Es=6,Ep=1,Ca=1,Cs=1,Cp=1,Ia=5,Is=4,alpha=0.6,beta=0.5,Ra=3,Rs=1,lamda=0.2,gamma=0.5,Fa=4,Fs=2,theta=0.5,Da=2,Ds=6;

[t,y]=ode45(@(t,y) zhubo(t,y,Ea,Es,Ep,Ca,Cs,Cp,Ia,Is,alpha,beta,lamda,Ra,Rs,theta,Fa,Fs,gamma,Da,Ds),[0 20],[0.5 0.5 0.5]);

points=1:1:length(t);

plot(t,y(:,2),'r','linewidth',1.5);

hold on;

Ea=2,Es=6,Ep=1,Ca=1,Cs=1,Cp=1,Ia=5,Is=4,alpha=0.6,beta=0.9,Ra=3,Rs=1,lamda=0.2,gamma=0.5,Fa=4,Fs=2,theta=0.5,Da=2,Ds=6;

[t,y]=ode45(@(t,y) zhubo(t,y,Ea,Es,Ep,Ca,Cs,Cp,Ia,Is,alpha,beta,lamda,Ra,Rs,theta,Fa,Fs,gamma,Da,Ds),[0 20],[0.5 0.5 0.5]);

points=1:1:length(t);

plot(t,y(:,2),'c','linewidth',1.5);

hold on;

set(0,'defaultfigurecolor','w')

grid on

hold on

xlabel('Time');

ylabel('Proportion(y)');

set(get(gca,'xlabel'),'FontName', 'Times New Roman','FontSize',12);

set(get(gca,'ylabel'),'FontName', 'Times New Roman','FontSize',12);

set(gca,'XTick',[0:4:20],'YTick',[0.3:0.1:1])

axis([0 20 0.3 1]);

legend('β=0.1', 'β=0.5 ', 'β=0.9');

figure(7c);

Ea=2,Es=6,Ep=1,Ca=1,Cs=1,Cp=1,Ia=5,Is=4,alpha=0.6,beta=0.1,Ra=3,Rs=1,lamda=0.2,gamma=0.5,Fa=4,Fs=2,theta=0.5,Da=2,Ds=6;

[t,y]=ode45(@(t,y) zhubo(t,y,Ea,Es,Ep,Ca,Cs,Cp,Ia,Is,alpha,beta,lamda,Ra,Rs,theta,Fa,Fs,gamma,Da,Ds),[0 10],[0.5 0.5 0.5]);

points=1:1:length(t);

plot(t,y(:,3),'b','linewidth',1.5);

hold on;

Ea=2,Es=6,Ep=1,Ca=1,Cs=1,Cp=1,Ia=5,Is=4,alpha=0.6,beta=0.5,Ra=3,Rs=1,lamda=0.2,gamma=0.5,Fa=4,Fs=2,theta=0.5,Da=2,Ds=6;

[t,y]=ode45(@(t,y) zhubo(t,y,Ea,Es,Ep,Ca,Cs,Cp,Ia,Is,alpha,beta,lamda,Ra,Rs,theta,Fa,Fs,gamma,Da,Ds),[0 10],[0.5 0.5 0.5]);

points=1:1:length(t);

plot(t,y(:,3),'r','linewidth',1.5);

hold on;

Ea=2,Es=6,Ep=1,Ca=1,Cs=1,Cp=1,Ia=5,Is=4,alpha=0.6,beta=0.9,Ra=3,Rs=1,lamda=0.2,gamma=0.5,Fa=4,Fs=2,theta=0.5,Da=2,Ds=6;

[t,y]=ode45(@(t,y) zhubo(t,y,Ea,Es,Ep,Ca,Cs,Cp,Ia,Is,alpha,beta,lamda,Ra,Rs,theta,Fa,Fs,gamma,Da,Ds),[0 10],[0.5 0.5 0.5]);

points=1:1:length(t);

plot(t,y(:,3),'c','linewidth',1.5);

hold on;

set(0,'defaultfigurecolor','w')

grid on

hold on

xlabel('Time');

ylabel('Proportion(z)');

set(get(gca,'xlabel'),'FontName', 'Times New Roman','FontSize',12);

set(get(gca,'ylabel'),'FontName', 'Times New Roman','FontSize',12);

set(gca,'XTick',[0:2:10],'YTick',[0.5:0.1:1])

axis([0 10 0.5 1]);

legend('β=0.1', 'β=0.5 ', 'β=0.9');
